# Targeting the gut microbiota and lipid metabolism: potential mechanisms of natural products for the treatment of non-alcoholic fatty liver disease

**DOI:** 10.3389/fphar.2025.1610498

**Published:** 2025-06-09

**Authors:** Yutian Zhang, Tianlin Wang, Junquan Han, Jielin Song, Chaoshuai Yang, Lei Liang, Huizhen Li, Hong Wang

**Affiliations:** ^1^ Graduate School, Tianjin University of Traditional Chinese Medicine, Tianjin, China; ^2^ Department of Gastroenterology, Tianjin University of Traditional Chinese Medicine Second Affiliated Hospital, Tianjin, China; ^3^ Department of General Surgery, Tianjin University of Traditional Chinese Medicine Second Affiliated Hospital, Tianjin, China

**Keywords:** gut microbiota, lipid metabolism, natural products, NAFLD, probiotics

## Abstract

Non-alcoholic fatty liver disease (NAFLD) is a chronic progressive liver disease with overnutrition and insulin resistance (IR) as the main etiologic factors. Hepatic lipid accumulation is a central factor contributing to this cascade of changes. Consequently, therapeutic interventions that target hepatic lipid metabolism and inflammatory response pathways hold considerable promise for the treatment of NAFLD. Furthermore, there is a close link between the gut microbiota (GM) and host health. GM and its metabolites can rely on multiple complex pathways to be deeply involved in the occurrence and development of NAFLD, which is associated with a variety of mechanisms. This makes it difficult to achieve satisfactory therapeutic efficacy of drugs targeting a single specific mechanism. In this context, natural products have the advantage of intervening in multiple targets and high safety. Consequently, an increasing number of researchers are considering natural products as a potential breakthrough point for the treatment of NAFLD. Notably, natural products influence intestinal mucosal permeability and metabolite production by regulating the abundance of beneficial flora in GM, which in turn regulates lipid metabolism to reduce hepatic steatosis and inhibit the progression of NAFLD. This paper reviews the research progress of natural products intervening in NAFLD through GM and its metabolites and lipid metabolism that has emerged in recent years, aiming to provide a basis for future natural product interventions in NAFLD.

## 1 Introduction

Non-alcoholic fatty liver disease (NAFLD), also known as metabolic dysfunction-associated fatty liver disease, MAFLD, is a term that has undergone a name change that has been advocated by multiple societies, led by the American Association for the Study of Liver Diseases, in 2023 (Rinella et al., 2023). This nomenclature change remains contentious due to its exclusion of patients with alcohol consumption, which is a significant proportion of individuals affected by fatty liver disease (Kim et al., 2023; Kokkorakis et al., 2023). This article still uses the old name, NAFLD.

The incidence of NAFLD exhibits geographical variation. Current global estimates posit that NAFLD affects 32.4% of the global population, with an escalating prevalence that is of significant concern on an annual basis (Riazi et al., 2022). NAFLD has the potential to progress to other liver diseases, such as NASH and irreversible liver fibrosis, cirrhosis and hepatocellular carcinoma (HCC). Additionally, it is closely related to cardiovascular and cerebrovascular diseases, metabolic syndrome, as well as chronic kidney disease (CKD) and a high incidence of extrahepatic malignancies (Thomas et al., 2024). This has a significant impact on the quality of life and long-term health of patients, and also places a considerable burden on the global healthcare system, suggesting the need for early intervention in NAFLD.

At present, Resmetirom is the only drug that has been approved by the FDA for the treatment of NASH, and it is notable that it can cause adverse effects (Keam, 2024). Concurrently, other clinical first-line drugs, such as SGLT-2 inhibitors, PPAR-γ agonists, GLP-1R agonists, and statins, while correcting the metabolic dysfunctions associated with NAFLD progression, also induce adverse effects including genitourinary infections, gastrointestinal reactions, worsening of heart failure, and osteoporosis (Hameed et al., 2023; Park et al., 2023; Wang Z. et al., 2023; Yang T. et al., 2024). In contrast, the therapeutic effects of vitamin E have been observed to be effective only in specific patient populations, including those possessing genetic variants of haptoglobin as well as genotypes of fatty acid desaturase 1/2 (FADS1/FADS2) (Banini et al., 2019). These approaches are insufficient to treat the increasing number of patients with NAFLD, and the urgent need exists to identify other effective therapeutic avenues.

Gut microbiota (GM) represents one of the most substantial microbial reservoirs within the human body (Pouwels et al., 2022), comprising approximately 10–100 trillion microorganisms in the gut of a typical adult (Younossi et al., 2018). These microorganisms play important roles in the processes of digestion and the maintenance of homeostasis of glucose/lipid metabolism (Paternostro and Trauner, 2022; Tilg et al., 2021). Dysregulation of GM has been demonstrated to result in disorders of glucose/lipid metabolism, inducing insulin resistance (IR) within the body, leading to abnormalities in fatty acids (FAs), triglyceride (TG), and cholesterol (TC), and causing hepatic steatosis. The metabolites of GM, such as bile acids (BAs), Short-chain fatty acids (SCFAs) and Trimethylamine N-oxide (TMAO), have been shown to be closely related to the energy metabolism of the organism (Caussy and Loomba, 2018). It is imperative to emphasise the significance of GM in the treatment of NAFLD. Traditional Chinese medicine (TCM) boasts numerous advantages, including multiple pathways of action, abundant targets, and low toxicity. TCM has demonstrated excellent potential in the treatment of NAFLD (Ji et al., 2022; Tan et al., 2023). Nevertheless, the absence of a definitive therapeutic mechanism hinders the advancement of TCM therapy for NAFLD. The exploration of natural products as a means to regulate lipid metabolism and intervene in NAFLD through GM and its metabolites is a promising avenue for further research.

## 2 Non-alcoholic fatty liver disease and dysfunctional lipid metabolism

The “multiple-hit” theory (Buzzetti et al., 2016) has gained widespread acceptance as the pathogenesis of NAFLD, proposing that the condition arises from the synergistic effect of environmental, dietary, lifestyle, epigenetic and other factors in individuals with a genetic predisposition (Juanola et al., 2021). The pathogenesis of NAFLD is the result of a combination of factors, but lipid metabolism disorders are still the core of NAFLD, and the liver, as an important lipid metabolising organ, greatly influences the lipid homeostasis in the organism (Böhm et al., 2013).

**FIGURE 1:**
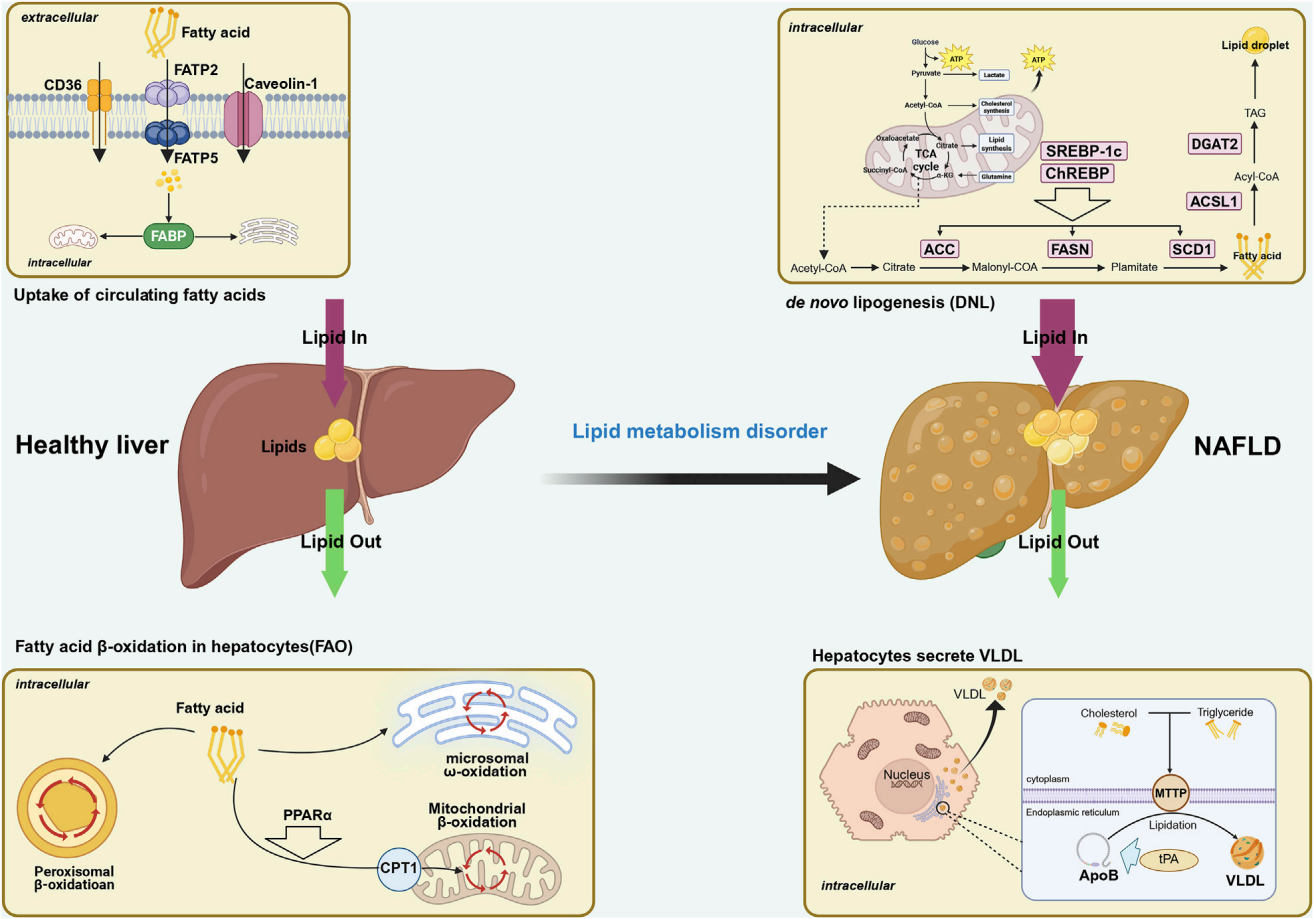
The thickness of the arrow represents the quantity in this figure. Possible mechanisms of the role of fatty acid metabolism in the development of NAFLD. Fatty acid metabolism is one of the important links in liver lipid metabolism, and its balance affects the progression of NAFLD. Fatty acid metabolism includes the uptake of circulating FFA, *de novo* lipogenesis (DNL), fatty acid β-oxidation (FAO), and the release of FFAs into the bloodstream in the form of very low-density lipoproteins (V-LDL). The first two increase the amount of fatty acids in the liver, while the latter two consume the amount of fatty acids in hepatocytes. When the increase in fatty acids is greater than the consumption, it will drive the development of NAFLD. NAFLD, non-alcoholic fatty liver disease; FATP, fatty acid transport protein; FABP, fatty acid-binding proteins; CAV-1, Caveolin-1; CD36, cluster of differentiation 36; FAO, fatty acid oxidation; TCA, tricarboxylic acid; FAS, fatty acid synthesis; DNL, *de novo* lipogenesis; ACC, acetyl-CoA carboxylase; FASN, fatty acid synthase; SCD1, stearoyl-CoA desaturase1; Dgat2, diacylgycerol acyltransferase; ACSL, acyl-CoA synthetaselong chain family member; PPARα, peroxisomeproliferator-activated receptor alpha; CPT1, carnitine palmitoyltransferase 1; MTTP, microsomal triglyceride transfer protein; tPA, tissue plasminogen activator; VLDL, very low density lipoprotein.

It is imperative to acknowledge the pivotal role of the balance between the rate of FAs accumulation and FAs degradation by hepatocytes in maintaining the low-fat state of the liver. The aforementioned balance encompasses the uptake of peripheral circulating free fatty acid (FFA), *de novo* lipogenesis (DNL), fatty acid oxidation (FAO), and entry into the bloodstream in the form of very low-density lipoproteins (V-LDL). These elements serve as the cornerstones for ensuring the balance of hepatic lipid metabolism (Paul et al., 2022). Conversely, an excess of FAs within hepatocytes leads to TG accumulation, which is a primary contributor to NAFLD (Santos-Baez and Ginsberg, 2021). A stable isotope tracer study (Tiwari and Siddiqi, 2012) demonstrated that the majority of TG accumulated in NAFLD (approximately 59%) originates from FFA produced by adipose tissue breakdown. Another significant source (Donnelly et al., 2005) is DNL synthesis (approximately 26.1%), and the remaining amount is derived from dietary intake (approximately 14.9%). This comprehensive analysis underscores the predominant role of FFA uptake from the circulation, along with NAL, as the pivotal source of TG accumulation within hepatocytes. As shown in Figure 1.

### 2.1 Key transporter proteins for free fatty acid uptake by the liver

Adipose tissue is the most significant TG storage site in the body. Stimulation of adipose tissue lipolysis to FAs results in entry into the peripheral circulation (Griffin et al., 2023). Hepatocytes rely on the uptake of FFA, with the uptake process mediated by fatty acid transporter proteins (FATP), fatty acid binding proteins (FABP), and human leukocyte differentiation antigen (CD36) (Canbay et al., 2007).

#### 2.1.1 Fatty acid transportation protein

Fatty acid transporter protein (FATP) is a class of transmembrane transporter proteins that are primarily responsible for transporting long-chain fatty acids from the extracellular to the intracellular environment. FATP2/5 is the isoform of FATP distributed on the mammalian liver and is responsible for the uptake of extracellular FFA in the hepatocytes. It was found that knockdown of *Fatp2* in mice reduced the ability of hepatocytes to uptake peripheral circulating FFA by 40%, and *Fatp2−/−* mice did not develop hepatic steatosis compared to normal mice that also consumed high-fat diet (HFD) and already had developed NAFLD (Falcon et al., 2010). Surprisingly, the knockdown of *Fatp5* also led to a reduction in the intrahepatic TG content, which was characterised by a decrease in the ability of hepatocytes to uptake long-chain FA and the activation of the NAL pathway (Doege et al., 2006). In comparison to normal subjects, FATP5 expression is notably elevated in the liver of NASH patients (Enooku et al., 2020), indicating that elevated FATP expression contributes to an increase in intrahepatic FA content, thereby promoting steatosis.

#### 2.1.2 Fatty acid binding protein

Fatty acid binding protein (FABP) is present in the liver in the form of Liver FABP (L-FABP), which functions to transport lipotoxic FFA to the mitochondria to participate in FAO or to the endoplasmic reticulum to participate in TG synthesis. This process serves to reduce the damage to hepatocytes caused by lipotoxicity (Spann et al., 2006). In the absence of *L-fabp*, a significant accumulation of lipids has been observed in the liver of mice (Martin et al., 2015). NAFLD patients exhibit elevated L-FABP expression levels, which gradually decline as the condition progresses, thereby diminishing the liver cells’ capacity to resist lipotoxicity. In comparison with the general population, NAFLD patients exhibit high specificity and sensitivity of L-FABP in the serum. Consequently, L-FABP in the serum has emerged as a promising diagnostic marker for NAFLD (Akbal et al., 2016). A positive correlation has been observed between the serum L-FABP levels and various indicators of NAFLD severity (Özenirler et al., 2013). This phenomenon may be attributed to L-FABP’s capacity to influence FAs metabolism through peroxisomeproliferator-activated receptor α (PPARα) (Pawlak et al., 2015) and expedite the progression of NAFLD by promoting steatosis and activating hepatic stellate cells (HSC) (Newberry et al., 2012; Chen et al., 2013). Research has indicated that serum L-FABP levels can serve as a marker of liver cell damage in patients with NAFLD (Tanoglu and Beyazit, 2016; Lu et al., 2020). Notably, serum L-FABP levels have also been shown to predict survival rates across various stages of chronic liver disease, including hepatitis, cirrhosis, and hepatocellular carcinoma (Eguchi and Iwasa, 2021). Furthermore, these levels have been observed to reflect the prognosis of hepatocellular carcinoma (HCC) of diverse etiologies (Eguchi et al., 2019).

#### 2.1.3 Cluster of differentiation 36

Cluster of differentiation 36 (CD36) is a translocase enzyme (FAT) that primarily facilitates the uptake of long-chain FAs. Under normal circumstances, CD36 is expressed at low levels in the liver (Su and Abumrad, 2009). However, an environment with high fat content has been observed to induce high expression of CD36 in the cytoplasm of liver cells. This expression is not only increased but also driven from the cytoplasm to the cell membrane (Chabowski et al., 2013). This, in turn, has been shown to exacerbate FAs metabolic disorders and induce liver inflammation (Zhao et al., 2018). CD36 serves as a crucial link between FAs and long-chain acyl-CoA synthetase (ACSL) (Zhao et al., 2018). Inhibiting CD36 palmitoylation has been shown to drive FAT localization in the mitochondria, thereby promoting fatty acid oxidation. HFD has been observed to increase CD36 palmitoylation in the liver of mice, which in turn reduces the transport of FAs to ACSL1, leading to increased lipid accumulation (Zeng S. et al., 2022). This underscores the notion that the inhibition of CD36 palmitoylation may serve as a therapeutic strategy to delay the progression of NAFLD. Additionally, obesity has been found to be closely associated with CD36. Ob/ob mice exhibit elevated CD36 protein levels in their livers (Nassir et al., 2013), and the CD36 content in the livers of patients with grade III obesity (BMI ≥35) is positively associated with liver fat content (Greco et al., 2008). Research studies have demonstrated that the amount of CD36 in the liver cells of NAFLD patients is higher than that observed in normal individuals. Furthermore, the expression of CD36 in the liver can enhance the uptake of FFA by liver cells, thereby leading to TG accumulation (Sheedfar et al., 2014; Zhang et al., 2018). Notably, the study (Zhong et al., 2017) revealed that the absence of *Cd36* does not impact the liver’s capacity for FFA uptake in murine models. Cardiomyocytes from subjects with *CD36* gene defects exhibited a complete loss of FFA uptake capacity due to the gene defect, while the uptake potential of liver cells was augmented (Yamashita et al., 2007). This evidence suggests that CD36 can drive the development and progression of NAFLD; however, the uptake of FFA by liver cells does not rely on CD36. CD36 is present in the peripheral circulation in the form of soluble CD36 (sCD36). Research studies (Handberg et al., 2012; Petta et al., 2013) have demonstrated that sCD36 can serve as a marker for the progression of fatty degeneration in the liver. The study (Rada et al., 2020) initially demonstrated that the plasma concentration of sCD36 can sensitively reflect the expression level of CD36 in the liver. Furthermore, an experiment using magnetic resonance spectroscopy to measure liver fat content (Heebøll et al., 2017) found that the concentration of circulating sCD36 was closely related to the level of intrahepatic lipids in NAFLD. Consequently, sCD36 in the blood emerges as a highly sensitive indicator of the severity of hepatocellular steatosis in patients with NAFLD.

#### 2.1.4 Caveolin-1

Caveolin-1 (CAV-1) is a structural protein of the caveolae (Jiang et al., 2023), which is involved in lipid metabolism by specifically binding to signalling molecules (Fernandes and Oliveira-Brett, 2020). Upregulation of CAV-1 expression effectively reduced TG levels in the peripheral circulation of a rat model of HFD and decreased lipid deposition in the liver, alleviating the progression of NAFLD (Deng et al., 2024). The mechanism by which CAV-1 interferes with hepatic lipid metabolism is not yet fully defined, but significant progress has been made in this area. Disturbed iron metabolism has been identified as a significant contributor to hepatocyte death in NAFLD, where the accumulation of Fe^2+^ within the cells results in the generation of substantial amounts of Reactive Oxygen Species (ROS) via the Fenton reaction, thereby initiating cell death (Teschke, 2022). CAV-1 activates the hepatocyte FTL/FTH pathway and drives the conversion of Fe^2+^ to Fe^3+^, which in turn inhibits oxidative stress in hepatocytes and ultimately alleviates liver injury during the course of NAFLD (Deng et al., 2023). CAV-1 inhibited the Akt/mtH pathway in the hepatocytes, and finally alleviated liver injury in the course of NAFLD. CAV-1 inhibited Akt/mtD, which was the most important factor in the metabolism of iron. CAV-1 has been shown to inhibit the Akt/mTOR pathway, thereby inducing lipid autophagy in NAFLD (Xue et al., 2020). In addition, the levels of Pink-1/Parkin content and autophagy-related proteins (LC3-II/I and Beclin-1) exhibited a positive correlation with CAV-1, while SREBP-1c content demonstrated a negative correlation with CAV-1 (Jiang et al., 2021). Upregulation of CAV-1 effectively activated the Pink-1/Parkin pathway-mediated mitochondrial autophagy, thereby inhibiting SREBP-1c expression and reducing cellular lipid accumulation. Researchers (Ding et al., 2018) successfully transfected plasmids overexpressing CAV-1 into HepG2 cells, thereby inducing an increase in intracellular TC efflux. Furthermore, *Cav-1* gene expression was found to be positively correlated with aortic endothelial cell ABCA1 levels, and negatively correlated with the level of cholesterol efflux from the aortic endothelial cells (Lin et al., 2007). This finding indicates that CAV-1 also affects cellular lipid metabolism by interfering with ABCA1 expression.

### 2.2 Hepatic *de novo* lipogenesis

Hepatic *de novo* lipogenesis (DNL) is another key mechanism for maintaining FA homeostasis in hepatocytes (Zeng H. et al., 2022), converting alternative carbon sources to FA through numerous enzymatic reactions, which are esterified and then stored in the liver as TG (Batchuluun et al., 2022). Typically, 2%–5% of the total amount of TG synthesized by the liver is derived from DNL (Diraison et al., 2003), and a high-carbon-water diet, obesity, and hyperinsulinemia increase this value to the 25%–30% range (Mk et al., 1993; Diraison et al., 1997; Siler et al., 1999), whereas starvation inhibits the DNL pathway (Cross et al., 2023). Consequently, the degree of DNL activity is closely related to the nutritional status of the organism. The study (Donnelly et al., 2005) utilised isotopes to examine the source of TG in the livers of patients with NAFLD, and found that 26% of the TG originated from the DNL pathway. The DNL pathway involves the conversion of acetyl-coenzyme A and malonyl-coenzyme A into fatty acids through a series of enzymatic reactions, including DNL, elongation, desaturation, and esterification (Hellerstein et al., 1996). Each step in the pathway is catalysed by specific enzymes, with the main enzymes responsible for *ab initio* synthesis being acetyl coenzyme A carboxylase (ACC) and fatty acid synthase (FAs) (Yue et al., 2018). Stearoyl coenzyme A desaturase 1 (SCD1) is the regulatory enzyme for lengthening and desaturation (Zheng et al., 2021), while diacylglycerol acyltransferase (DGAT) and long-chain acetyl coenzyme A synthase 1 (ACSL1) are the regulatory enzymes for the esterification step (Filali-Mouncef et al., 2022). The process is primarily regulated by two key transcription factors, sterol regulatory element binding protein 1c (SREBP 1c) and carbohydrate regulatory element binding protein (ChREBP) (Linden et al., 2018), which are induced by insulin and glucose, respectively (Kawano and Cohen, 2013; Oosterveer and Schoonjans, 2014). Consequently, the present study aimed to review the effects of the DNL pathway on hepatic lipid metabolism, with a view to exploring clinical strategies for treating NAFLD by interfering with NAL.

#### 2.2.1 Key transcription factors in *de novo* lipogenesis

##### 2.2.1.1 SREBP-1c

SREBP-1c is one of the three SREBP isoforms (1a, 1c, 2) present in mammals (Eberlé et al., 2004). SREBP-1c is predominantly found in the liver and is exclusively responsible for the regulation of hepatic FA synthesis (Shimano and Sato, 2017). SREBP cleavage-activating protein (SCAP) is essential for activating the transcriptional activity of *Srebp* (Matsuda et al., 2001). Researchers (Horton et al., 2003) found that disrupting the transcriptional activity of SREBP by knocking out the *Scap* gene resulted in a near loss of lipid synthesis in mouse liver. The study (Jiang et al., 2022) exploited the fact that 25-hydroxyalcohol (25-HL) has a greater ability to sequester SCAP-SREBP, and by binding to insulin-inducible gene (INSIG) proteins, induced the coupling of INSIG to SCAP, resulting in the SREBP retention in the endoplasmic reticulum and inability to activate it, which in turn inhibits hepatic lipogenesis. These findings underscore the pivotal role of SREBP in hepatic lipid synthesis. As a member of the SREBP isoforms primarily implicated in hepatic FA synthesis, SREBP-1c activates the transcription of ACC1, FAS, and SCD1, thereby stimulating the DNL pathway and leading to the production of substantial quantities of FA, resulting in hepatic steatosis (Choi et al., 2014). SREBP-1c is closely associated with the progression of hepatic NAFLD (Badmus et al., 2022). SREBP-1c overexpression has been demonstrated to trigger hepatocyte lipid accumulation (Shimano et al., 1997). The hepatic deletion of SREBP-1c protein in ob/ob mice resulted in a 50% decrease in intrahepatic TG (Moon et al., 2012). Downregulation of SREBP-1c levels in mice by using antisense oligonucleotides was effective in reversing hepatic steatosis induced by HFD (Vitto et al., 2012). In addition, patatin-like phospholipase structural domain protein 3 (PNPLA3), which is closely related to NAFLD, can contribute to hepatic steatosis in several ways (Ericson et al., 2022). SREBP-1c upregulates the increased expression of the *Pnpla3* gene by binding to the PNPLA3 promoter, which in turn promotes lipid accumulation in the liver (Qiao et al., 2011). Furthermore, endoplasmic reticulum stress has been demonstrated to activate SREBP-1c (Ferré et al., 2021). The activator of transcription factor 6 (AFT6), the principal sensor of endoplasmic reticulum stress, exhibits analogous activation conditions to SREBP-1 (Ye et al., 2000). During endoplasmic reticulum stress, activated AFT6 activates SREBP-1c via the PERK-IRE1-eIF2α-ATF6 pathway, which in turn drives hepatocyte steatosis (Lee et al., 2012; Röhrl et al., 2014).

##### 2.2.1.2 Carbohydrate regulatory element binding protein

Carbohydrate regulatory element binding protein (ChREBP) is a major regulator of DNL in the liver and is involved in glycolysis (Ishii et al., 2004), regulating the conversion of glucose to FA via the DNL pathway (Postic et al., 2007). ChREBP acts as a major glucose-responsive transcription factor (Yamashita et al., 2001), and high glucose status promotes the translocation of ChREBP into the nucleus and increases transcriptional activity (Li et al., 2006). It has been established that glucose, fructose, and even glucose derivatives (Iizuka et al., 2004) activate ChREBP expression. In turn, ChREBP is able to activate the expression of enzyme genes associated with DNL, such as ACC, FAS, and SCD1, thereby promoting lipid synthesis in the liver (Ishii et al., 2004). In addition, ChREBP is also involved in the maintenance of glucose homeostasis. To test the hypothesis that ChREBP deficiency causes a decrease in insulin sensitivity, researchers used a hyperinsulinemic euglycemic clamp to test insulin sensitivity in Liver-*Chrebp* KO mice (Jois et al., 2017). They found that only a reduction in exogenous glucose input ensured that the mice had blood glucose at basal levels, suggesting that *Chrebp* deficiency caused a decrease in insulin sensitivity.

Furthermore, the selective knockdown of *Chrebp* in hepatocytes of ob/ob mice significantly reduces lipid accumulation in hepatocytes and alleviates TG and FFA levels in the peripheral circulation (Dentin et al., 2006). This finding suggests that *CHREBP* knockdown is effective in reducing hepatic lipid accumulation by the DNL pathway. However, it should be noted that this does not necessarily imply that knockdown of *Chrebp* alone is beneficial to the organism. ChREBP also affects fibroblast growth hormone 21 (FGF21) expression in the liver (Iizuka et al., 2009).

The latter has been demonstrated to inhibit the body’s sweet taste preference as well as sugar intake by acting on glutamatergic neurons in the ventral medial hypothalamus (Jensen-Cody et al., 2020). Moreover, ChREBP has been shown to promote the ubiquitination and subsequent degradation of nSREBP2, which in turn inhibits the biosynthesis of TC (Luo et al., 2020). ChREBP, a major component of the DNL pathway, which is responsible for the conversion of sugars into fats, is involved in a number of complex biological activities. While the knockdown of *Chrebp* can reduce the FA generated by the DNL pathway, it can also lead to other problems, indicating that direct inhibition/knockdown of *Chrebp* is not an effective solution to hepatic lipid accumulation.

#### 2.2.2 Redirected synthesis of important regulatory enzymes

##### 2.2.2.1 Acetyl coenzyme a carboxylase

Acetyl coenzyme A carboxylase (ACC) is the rate-limiting step in FA anabolism (Wang et al., 2022) and is biologically dependent (Packman and Whitney, 1990). Two isoforms of ACC have been identified in humans: ACC1 and ACC2 (Brownsey et al., 2006). The most significant difference between them is that ACC2 possesses an additional amino-terminal hydrophobic sequence, which is responsible for its ability to specifically anchor to the outer mitochondrial membrane (Abu-Elheiga et al., 2000). It has been established (Bianchi et al., 1990; Kim, 1997) that ACC1 functions as the rate-limiting enzyme of the DNL process, localised in the cytoplasm and predominantly distributed in adipogenic tissues (including liver and adipose) (Kreuz et al., 2009). ACC2 is located in the mitochondrial membrane and is primarily responsible for the regulation of FAO, and the malonyl-coenzyme A variant produced by ACC2 has been shown to inhibit the activity of carnosine palmitoyltransferase 1 (CPT-1). This, in turn, inhibits the LCFA-CoAs transport to the mitochondria via CPT1 to participate in FAO (Hoy et al., 2021). Furthermore, the inhibition of ACC has been shown to alleviate hepatocellular lipid accumulation by down-regulating DNL as well as promoting FAO (Bourbeau and Bartberger, 2015). The study (Ross et al., 2020) found that oral administration of a hepatic ACC1/ACC2-targeted inhibitor (PF-05221304) to mice in a Western dietary model inhibited intrahepatic DNL, attenuated hepatic steatosis, and inhibited the activation process of hepatic stellate cells shifting to fibroblasts. In an experiment (Bates et al., 2020) using other ACC inhibitors (FIR) to intervene in HepG2 cells and mice, researchers found that the use of FIR was effective in reducing the DNL pathway and concomitantly augmenting FAO, and that this change was observed in in vivo and *in vitro* experiments. However, the opposite result of ACC deletion has also been observed, and it has been reported (Loomba et al., 2018) that deletion of ACC elevates circulating TG levels. The study found that knockdown of *Acc* significantly elevated plasma TG levels (200%) (Kim et al., 2017), and that *Acc* knockdown decreases the concentration of PUFA and thereby increases SREBP-1 activity, whereas restored-activated SREBP-1 catalyzes TG by activating the GPAT1 to catalyze TG synthesis and promote VLDL secretion into the circulation to trigger hyperlipidemia. *Acc−/−* mice with decreased lipoprotein lipase (LPL) activity have reduced TG clearance leading to hyperlipidemia (Goedeke et al., 2018). *ACC* deletion has been shown to inhibit PPARα expression, which enhances LPL activity, and therefore, in order to avoid adverse effects, knockdown of *ACC* to treat NAFLD may need to be coupled with PPARα agonists. Clinical trials (Calle et al., 2021) also observed that ACC inhibitors elevated TG levels in patients’ plasma, but the combination of lipid-lowering drugs/PPARα agonists would resolve the TG elevation associated with ACC inhibitors. Consequently, further discourse is necessary to ascertain whether *ACC* knockdown holds potential benefits for NAFLD patients.

##### 2.2.2.2 Fatty acid synthase

Fatty acid synthase (FASN) is a protein composed of seven subunits (Long and Cravatt, 2011), which is responsible for catalyzing the synthesis of palmitic acid (PA) from acetyl coenzyme A and malonyl coenzyme A in a 7:1 ratio during DNL, and PA is then extended by very long chain fatty acid elongase 6 (ELOVL6) and desaturated by stearoyl coenzyme A desaturase 1 (SCD1) to produce oleic acid (Parlati et al., 2021). *Fasn* transcription is predominantly subject to regulation by SREBP1c (Postic and Girard, 2008). The feeding of a high-fat, high-sucrose diet (HFD) to liver-*Fasn* KO mice has been demonstrated to cause the development of hepatic steatosis (Chakravarthy et al., 2005). The high expression of FASN in the liver (Dorn et al., 2010) has been shown to result in the accumulation of malonyl coenzyme A, thereby inhibiting FAO. FASN has been identified as the rate-limiting enzyme in the final step of FA synthesis by the DNL pathway (Nguyen et al., 2008), which exerts a significant influence on the upper limit of the hepatic capacity of the FA derived from the DNL pathway (Dorn et al., 2010). Researchers (Zhang et al., 2020) used MicroRNA-103 to target and inhibit the expression of FASN, which effectively inhibited FA synthesis via the DNL pathway and attenuated hepatic lipid accumulation. FASN was also associated with bioIR, one of the high-risk factors for NAFLD (Chen et al., 2023). In the DNL pathway, FASN catalyses the production of palmitic acid (PA), diglycerides (DAG), and ceramides, which activate protein kinase C (PKC) and damage mitochondria and the endoplasmic reticulum through inhibition of phosphorylation of the IRS1/PI3K site (Zhou et al., 2022), ultimately causing IR (Palomer et al., 2018). Related experiments have also demonstrated that inducing ubiquitinated degradation of FASN effectively ameliorates hepatic lipid accumulation in NAFLD mice (Xu et al., 2024). This finding suggests that the inhibition of FASN may represent a promising therapeutic approach for the management of NAFLD. However, the knockdown of *Fasn* has been observed to result in a decrease in PA content (Kang et al., 2024). It has been established that PA activates inflammation through the TLR4-NFκB pathway in HSC cells and upregulates the expression of pro-fibrotic genes, exacerbating MASH progression (Dong et al., 2020).

##### 2.2.2.3 Stearoyl coenzyme a desaturase 1

Stearoyl coenzyme A desaturase 1 (SCD1) is located in the endoplasmic reticulum (Heinemann and Ozols, 2003). SCD1 feeds DNL by converting saturated fatty acids (SFAs) to monounsaturated fatty acids (MUFAs), and is a key rate-limiting enzyme for DNL (Flowers and Ntambi, 2008). Deletion of the *Scd1* gene has been shown to inhibit TG production by the DNL pathway and to upregulate liver and brown adipose (BAT) cell oxidation (Dobrzyn et al., 2004), enhancing body thermogenesis (Lee et al., 2004). The knockdown of *Scd1* has been shown to inhibit ceramide biosynthesis (Dobrzyn et al., 2005), primarily due to the fact that SCD1 deletion causes a decrease in the expression level and activity of a key enzyme (serine palmitoyltransferase) required for ceramide synthesis, and a decrease in the synthesis of the substrate (palmitate) (Wang K. et al., 2020). The accumulation of ceramide has been demonstrated to induce lipotoxicity (Unger, 2002), whilst concurrently promoting lipid synthesis in hepatocytes (Wang et al., 2024). SCD1 deficiency has been observed to promote the phosphorylation of AMP in combination with AMPK (Blázquez et al., 2001), which in turn reduces malonyl coenzyme A synthesis by inhibiting ACC. This, in turn, has been shown to increase CPT1 activity and facilitate the transport of FA to the mitochondria to participate in FAO (Longo et al., 2019). In summary, the suppression of SCD1 expression has been shown to inhibit the expression of genes involved in DNL while concomitantly upregulating the expression of genes associated with FAO (Ntambi et al., 2002). However, it should be noted that this does not automatically imply that the suppression of SCD1 is beneficial to human health. It is important to note that excess lipids can contribute to the development of various metabolic diseases; however, essential lipids remain vital components of the body’s biometabolism (Sen et al., 2013). A study (Piccinin et al., 2019) found that the maintenance of the health of Liver-*Scd1*-KO mice is dependent on the dietary supplementation of oleic acid deficiency caused by SCD1 deletion, without which the body may suffer severe liver injury. Although SCD1 deletion inhibits the synthesis of TGs, it also leads to insufficient synthesis of MUFA as well as the accumulation of SFA, which in turn leads to ER stress and inflammation, and ultimately, to liver injury (Flowers et al., 2006; 2008). It has been established (Rizki et al., 2006) that MUFA synthesised by SCD1 in the DNL pathway confers a protective effect on the liver in numerous instances. This is attributable to the fact that the absence of SCD1 results in the accumulation of lipids that are more toxic than MUFA in the liver. Conversely, the supplementation of SCD1 has been shown to reduce the amount of lipids with greater toxicity in the liver (Piccinin et al., 2019). In addition, SCD1 protects the liver by inhibiting iron death, and SCD1 inhibits iron death by down-regulating lipid peroxide production that induces iron death, which promotes NAFLD (Liu et al., 2021; Chen et al., 2022). The relationship between SCD1 and iron death may be a novel target for the future treatment of NAFLD.

##### 2.2.2.4 Diacylglycerol acyl-transferase 2

Diacylglycerol acyl-transferase 2 (DGAT2) is the catalytic enzyme for the final step in the conversion of diacylglycerol to TAG, and includes two isoforms, DGAT1 and DGAT2 (Yen et al., 2008). DGAT2, which is abundantly expressed in the liver, primarily uses fatty acids from the DNL pathway to synthesize TG (Parlati et al., 2021), and researchers (Gluchowski et al., 2019) found that *Dgat2* deletion downregulated hepatic expression of DNL-related genes and significantly reduced hepatic TAG levels (by 70%) in NAFLD mice. The whole-body TG content of *Dgat2−/−* mice was only 10% of that of wild-type mice, with almost undetectable TG concentrations in the liver (Stone et al., 2004). The present study investigates the efficacy of specific knockdown of *Dgat2* in the liver of ob/ob mice in reducing NAFLD severity (Chen et al., 2002). These results suggest that the inhibition of DGAT2 may represent a significant intervention strategy for NAFLD, given its ability to influence TG synthesis through multiple pathways. Firstly, the inhibition of DGAT2 expression has been demonstrated to impede the TG esterification process. Secondly, DGAT2 deficiency has been shown to decrease the level of SREBP-1c transcription (Rong et al., 2024), which is responsible for FA synthesis. It is noteworthy that SREBP-2 remains unaffected in these circumstances. Since SREBP is initially localized to the endoplasmic reticulum membrane, it binds to SREBP cleavage-activating protein (SCAP) to form a stable complex, which is cleaved in order to form a mature SREBP. Inhibition of DGAT2 caused phosphatidylethanolamine (PE) enrichment in the endoplasmic reticulum (ER), blocking the cleavage of SREBP-1 independently of Insigs, which in turn inhibited SREBP-1 activation and suppressed TG synthesis by hepatocytes via the DNL pathway (Rong et al., 2024).

##### 2.2.2.5 Acyl-CoA synthetase long chain family member 1

Acyl-CoA synthetase long chain family member (ACSL) plays a crucial role in fatty acid metabolism and lipid homeostasis by catalyzing the synthesis of acyl coenzyme A (Acyl-CoAs) from FFA. There are five different isoforms of ACSLs in the human body, of which ACSL1 is the predominant isoform, contributing 50% of the hepatic ACSLs activity (Dong et al., 2023). The subcellular location of ACSL1 dictates its function (Soupene and Kuypers, 2008). When localized in the mitochondria, ACSL1 facilitates the role of acyl-CoAs in fatty acid oxidation (FAO). Conversely, when ACSL1 is localized in the endoplasmic reticulum, it contributes to the TANK-binding kinase 1 (TBK1) (Huh et al., 2020). TBK1 is a serine/threonine protein that acts as an effector of inflammatory signaling in adipocytes and hepatocytes. In addition, TBK1 functions as a scaffolding protein that binds to ACSL1, thereby driving ACSL1 localization to mitochondria to enhance FAO. A study on Alzheimer’s disease (AD) (Haney et al., 2024) found that *ACSL1* is the most important lipid synthesis gene for the formation of LD from TG in microglia in brain tissue, and overexpression of ACSL1 induced the synthesis of LD from TG in brain tissue, and the inhibition of ACSL1 attenuated the accumulation of LD in brain tissue, but further studies are needed to find out whether it also has such a role in the liver. Sortilin, a key regulator of the subcellular distribution of ACSL1 (Yang M. et al., 2024), has been shown to promote the translocation of mitochondrial ACSL1 to the nuclear endosome/lysosome. In addition, Consumption of sortilin has been observed to increase mitochondrial ACSL1 in adipocytes, thereby promoting the browning of white adipose tissue (WAT) and, consequently, reducing hepatic lipid deposition (Stanford et al., 2013). Lysine acetylation has been identified as a regulatory mark in almost all enzymes involved in FA anabolism (Zhao et al., 2010), and site mutation experiments have confirmed that acetylation at the specific sites K407 and K425 on the ACSL1 protein enhances its enzyme activity (Frahm et al., 2011; Chen Z. et al., 2018). However, there have been no experimental studies investigating the effect of acetylated ACSL1 on the NAFLD effects. The present study hypothesises that ACSL1 acetylation can be regulated by SIRT to enhance ACSL1 activity, to promote FAO, and ultimately affect NAFLD progression. This may be a novel strategy for future intervention in NAFLD.

### 2.3 Fatty acid oxidation in hepatocytes

Fatty acid oxidation (FAO) is accomplished intracellularly in mitochondria, peroxisomes, and microsomes on the endoplasmic reticulum (ER) (Dixon et al., 2021). It is important to note that there is variability in the FAs, as well as the catalytic enzymes involved in FAO at different subcellular levels. Mitochondria are the most prominent site of FAO (Adeva-Andany et al., 2019). In this process, FA is initially activated in the cytosol by lipoyl coenzyme A synthase, resulting in the formation of lipoyl coenzyme A. Carnitine palmitoyltransferase 1 (CPT1) then traps this lipoyl coenzyme A, forming lipoyl carnitine, which subsequently contributes to the process of FAO (Neuschwander-Tetri, 2010). CPT1 has been identified as the key rate-limiting enzyme in the mitochondrial FAO pathway (Fontaine et al., 2012), and it has been demonstrated that interference with the translocation function of CPT-1 can inhibit FAO (Abu-Elheiga et al., 2000).

Researchers (Weber et al., 2020) found that adeno-associated virus serotype 9 (AAV9) is the most potent AAV in gene therapy targeting the liver, and combining AAV9 with a heterodimer of human CPT1A (hCPT1a.m.) to form AAV9-hCPT1a.m., and injecting AAV9-hCPT1a.m. intravenously into the tails of mice with a model of NAFLD, this resulted in a significant increase in liver fatty acid oxidation (FAO) and a reduction in hepatic steatosis induced by HFD. The observed outcomes may be attributed to the ability of AAV9-hCPT1a.m. to generate mutants that enhance CPT1 activity in the mouse liver. Enhancement of CPT1 is effective in promoting FAO and thus attenuating hepatic lipid accumulation. However, this does not imply that enhancing CPT1 expression is an effective strategy for treating NAFLD. Study (Fondevila et al., 2022) found that CPT1A was highly expressed in patients with liver fibrosis and activated HSC in mice, which was positively correlated with the degree of liver fibrosis, and in fibrotic hepatocytes, CPT1A overexpression increased FAO, which stimulated the production of ROS, and ultimately the activation of HSC, whereas the inhibition/specific knockdown of *CPT1* blocked the activation of HSC, which then interfered with the progression of liver fibrosis. In summary, in early NAFLD, promoting CPT1 expression helps to promote FAO to reduce intrahepatic lipids, while enhancing CPT1 accelerates the process of hepatic fibrosis when NAFLD shifts to hepatic fibrosis, thus intervening CPT1 at different stages of NALFD may reap completely opposite results.

### 2.4 Very low density lipoprotein secretion by hepatocytes

FA that is not utilised by FAO is esterified to TG, which is subsequently exported from the liver as very low density lipoprotein (VLDL). In this process, apolipoprotein B (ApoB) is the structural scaffold on which VLDL is built. During ApoB lipidation, VLDL translocates TG, TC, and phospholipids to ApoB by virtue of microsomal triglyceride transfer protein (MTTP) to assemble into spherical particles (Hussain et al., 2012). ApoB in turn secretes assembled VLDL into the circulation (Sparks et al., 2011).

MTTP plays a crucial role in the process of ApoB lipidation, a process which is essential for the acquisition of lipoprotein biosynthetic function and stability. In the absence of MTTP, the unique sequence features of ApoB render it susceptible to reversal of translocation and subsequent proteasomal degradation (Zhang et al., 2025). *Mttp* ± mice were fed a standard diet, and oil red O staining of their livers revealed the presence of numerous intracellular lipid droplets in liver cells (Hussain and Bakillah, 2008). Liver-*Mttp*-KO mice exhibited a 40% decrease in serum TG content and a 50% decrease in TC content compared to WT mice, despite increased liver TG and TC content and hepatocyte-enriched lipid droplets (Hussain et al., 2012). In addition, MTTP-mediated ApoB lipidation is also subject to regulation by intracellular tissue-type plasminogen activator of fibrinolysis (tPA/PLAT), and intrahepatocyte tPA does not affect MTTP protein expression levels (Dai et al., 2023). tPA acts directly on ApoB to block the ApoB-MTP interaction, thereby inhibiting MTTP-mediated neutral lipid transfer and ApoB lipidation. Hepatocyte tPA expression has been shown to be negatively correlated with TC and TG concentrations in mouse serum (Dai et al., 2023). Plasminogen activator inhibitor 1 (PAI-1) is a serine protease inhibitor that binds to tPA in hepatocytes. PAI-1 binds to hepatocyte tPA, blocks the inhibitory effect of tPA on ApoB, and promotes the assembly and secretion of VLDL. In summary, interfering with MTTP-mediated VLDL assembly and secretion based on the interaction between tPA, PAI-1, and apoB not only interferes with atherosclerotic cardiovascular disease (CVD), but may also be a potential new strategy for the treatment of NAFLD.

## 3 Crosstalk between gut microbiota, and its metabolites, and lipid metabolism pathways in non-alcoholic fatty liver disease

**FIGURE 2:**
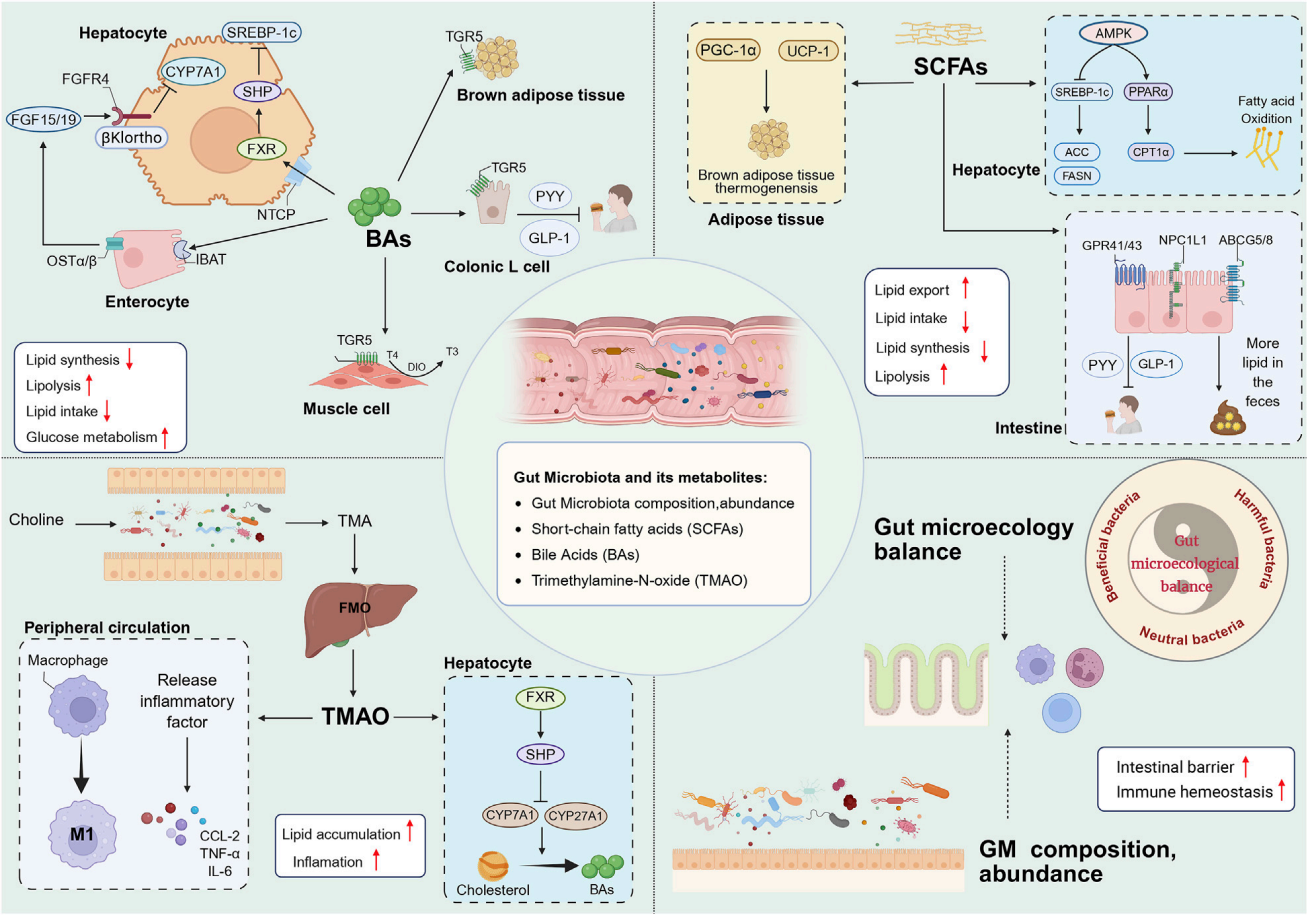
BAs contribute to host metabolism in various organs through FXR and TGR5. BAs synthesis in the liver and glucose metabolism are regulated by the intestinal FXR-FGF15/19 signal, and BAs also affect lipid synthesis in the liver through the FXR-SHP signal pathway. In addition, BAs enhance host energy metabolism through TGR5, including driving BAT thermogenesis; promoting the conversion of inactive thyroxine (T4) to active thyroid hormone (T3) in skeletal muscle to increase energy consumption; and promoting the release of GLP-1 and PYY by colon L cells to improve IR and suppress appetite. SCFAs promote BAT thermogenesis by activating PGC-1α and UCP-1 in adipose tissue. Secondly, SCFAs activate AMPK in the liver, on the one hand, SCFAs can downregulate DNL by inhibiting SREBP-1c and thereby reducing the expression of ACC and FASN, and on the other hand, they can promote FAO by activating PPARα and thereby upregulating the expression of CPT1α. In addition, SCFAs can reduce intake by activating GPR41/43 in the intestine to release PYY and GLP-1, and reduce lipid uptake through ABCG5/8 and NPC1L1, thereby increasing lipid excretion in the feces. TMAO enhances the pro-inflammatory polarization of macrophages and the release of inflammatory factors. TMAO inhibits the conversion of TC to BAs through the liver FXR/SHP signaling pathway. The balance of the intestinal microecology and the diversity of GM help to stabilize the intestinal mucosal barrier and immune system. BAs, bile acids; FXR, farnesoid X receptor; TGR5, G proteincoupled bile acid receptor; FGF15/19, fibroblast growth factor 15/19; FGFR4, fibroblast growth factor receptor 4; NTCP, sodium dependent taurocholate co-transporting polypeptide; OSTα/β, organic solute transporter subunit α/β; DIO2, Type II iodothyronine deionidinase; SHP, small heterodimer partner; BAT, brown adipose tissue; GLP-1, glucagon-like peptide-1; PYY, peptide YY; SCFAs, short chain fatty acid; PGC-1α, Peroxisome proliferator-activated receptor-γ coactivator-1α; UCP-1, Uncoupling protein 1; AMPK, AMP-activated protein kinase; SREBP-1c, sterol regulatory element-binding protein-1c; ACC, acetyl-co carboxylase; FASN, fatty acid synthase; PPARα, Peroxisome proliferator-activated receptor α; CPT1α, carnitine palmitoyltransferase-1 α; GPR41/43, G protein-coupled receptors 41/43; ABCG5/8, ATP-binding cassette transporter G5/8; NPC1L1, Niemann-Pick type C1 like1; TMAO, Trimethylamine oxide; TC, cholesterol; GM, gut microbiota.

The GM constitutes the largest microbial population in the human body, comprising approximately 100 trillion microorganisms. The GM maintains host metabolic homeostasis by consuming exogenous food or endogenous host substances to produce a variety of metabolites, which in turn interact with the host. The bidirectional interaction between the GM and the liver is termed the gut-hepatic axis, and the two interact with each other via the portal circulation. Alterations in GM composition, function, and metabolite profiles have the capacity to disrupt host-microbe homeostasis (Wu et al., 2021). GM dysregulation has been shown to result in increased gastrointestinal permeability, lipopolysaccharide translocation, immune activation, and altered BAs signalling, which in turn contributes to the development of NAFLD, MASH (Anstee et al., 2019). The portal circulation facilitates the entry of toxic substances produced by the GM into the liver, thereby exposing it to the metabolites generated by the gut microbiota. This has been shown to be a direct trigger for metabolic disorders and degenerative necrosis of hepatocytes (Song and Zhang, 2022). A range of colony-specific metabolites (including BAs, SCFAs, branched-chain amino acids, TAMO) have been implicated in the pathogenesis of metabolic disorders (Bauer et al., 2022). Intervention with GM and its metabolites has also been recognised as an important breakthrough for targeted therapy of NAFLD (A et al., 2021). As shown in Figure 2.

### 3.1 Gut microbiota composition, abundance changes and non-alcoholic fatty liver disease

Four of the most common types of bacteria are present in the intestinal tract of healthy adults, including *Firmicutes*, *Bacteroidetes*, *Actinobacteria*, and *Proteobacteria* are the most dominant bacterial phyla in the human gut (Shapira, 2016). With respect to abundance, the *Bacteroidetes* and *Firmicutes* are the most prevalent, followed by *Proteobacteria*, *Fusobacteria*, *Tenericutes*, *Actinobacteria* and *Verrucomicrobia*. Collectively, they constitute 90% of the total human gut microbiota (Gomaa, 2020). GM are dynamically changing collections of communities, and these microbial communities are correlated with the host’s age, health status, diet, and lifestyle. A significant disparity in the compositional structure, as well as the abundance of GM, has been observed between patients with NAFLD and healthy populations, and this discrepancy has been termed intestinal microecological dysbiosis (Quesada-Vázquez et al., 2022). In a seminal study, GM from healthy and NAFLD mice was transplanted into the intestines of two groups of germ-free mice. The results indicated that GM from NAFLD mouse sources elevated the risk of NAFLD in germ-free mice (Le Roy et al., 2013). The compositional structure and abundance of GM are differentially characterised at different stages of NAFLD (Mouzaki et al., 2013).

Researchers conducted a comparative analysis of the GM of NAFLD patients and the normal population (Spencer et al., 2011). They revealed that the GM of NAFLD patients exhibited a higher abundance of Gram-negative bacteria, with the abundance of the *Bacteroidetes*, which belongs to Gram-negative bacteria, increasing by 20%, while the abundance of *Firmicutes*, a predominant member of Gram-positive bacteria, decreased by 24%. The ratio of *Bacteroidetes* to *Firmicutes* is elevated in NAFLD patients (Wang et al., 2016). Specifically, the relative abundance of *Ruminococcaceae*, which are known to produce SCFAs as part of *Firmicutes*, exhibited a marked reduction in GM. The abundance of *Escherichia*, *Prevotella*, and *Streptococcus* increased, while *Faecalibacterium, Clostridium, Bacteroides and Lactobacillus* are lacking in NAFLD patients (Li et al., 2021). In patients with MASH, the proportion of *Clostridium coccoides* in the intestine was found to be significantly higher (Zhu et al., 2013). The severity of NAFLD is closely related to GM. For instance, an elevated abundance of *Bacteroides* has been identified as an independent risk factor for the severity of NASH, while the progression of liver fibrosis is closely associated with the presence of *Ruminococcus* (Boursier et al., 2016). In patients with liver fibrosis, a decline in the numbers of *Enterococcus faecalis* and *Faecalibacterium prausnitzii* (*F. prausnitzii*) has been observed, and the butyrate produced by these two flora has been shown to contribute to the maintenance of intestinal barrier function (Kwan et al., 2022).

In addition, GM can influence NAFLD through BAs metabolism. It is well known that microbial modifications of GM origin are essential for enterohepatic recycling of BAs, and that the synthesis of BAs, the size and compositional structure of the bile acid pool are dependent on GM.3-succinimidylated cholic acid (3-sucCA) is the primary bile acid, one of the types of BAs (CA), mainly from *Bacteroides uniformis*, and 3-sucCA levels are negatively correlated with NAFLD severity (Nie et al., 2024). Supplementation with *Bacteroides uniformis* has been shown to significantly ameliorate hepatic steatosis, as well as the degree of inflammation and fibrosis in MASH mice. A study (Leung et al., 2022) was conducted in which metagenomic metabolomics analysis was performed on fecal samples from 90 patients with NAFLD and 90 healthy individuals. The results indicated that, at the genus level, *Methanobrevibacter*, *Phascolarctobacterium*, and *Slackia* were independent risk factors for NAFLD, independent of obesity. At the species level, *Dorea formicigenerans* (*D. Formicigenerans*) was identified as an independent risk factor for NAFLD, independent of obesity. Gut barrier dysfunction has been identified as a pivotal factor in the progression of NAFLD (Lechner et al., 2020), characterised by the disruption of the intestinal mucosal barrier, thereby facilitating the passage of deleterious substances such as GM metabolites, bacteria, and enterogenous LPS through the portal system. This, in turn, results in the exacerbation of the hepatic inflammatory response, leading to liver injury and fibrosis (Ferro et al., 2020). Consequently, the modulation of the gut microbiota has emerged as a promising therapeutic approach for NAFLD management (Li et al., 2022). The restoration of GM ecological balance through probiotics, prebiotics, and fecal microbiota transplantation (FMT) has emerged as a novel therapeutic strategy to enhance NAFLD treatment (Carpi et al., 2022). In addition, engineered bacteria have emerged as a novel class of biotherapeutics, wherein the genetic material of bacteria is deliberately modified through genetic engineering to generate bacterial metabolites that are conducive to the control of disease progression (Canale et al., 2021). The experimental findings support these propositions, as researchers discovered that supplementation of probiotics to the gut significantly suppressed hepatic steatosis as well as intestinal inflammation in NAFLD mice (Ma et al., 2013). A meta-analysis (Sharpton et al., 2019) encompassing 1,252 patients also determined that supplementation with probiotics or synbiotics exhibited a strong correlation with enhanced liver function, diminished liver stiffness values (LSM), and the alleviation of hepatic steatosis in patients diagnosed with NAFLD.

### 3.2 Gut microbiota metabolites

#### 3.2.1 Trimethylamine N-oxide

Trimethylamine N-oxide (TMAO) is primarily derived from dietary choline, which is converted to trimethylamine (TMA) via the GM and subsequently generates TMAO by hepatic plus monooxygenase enzymes (FMOs) (Subramaniam and Fletcher, 2018). TMAO drives NAFLD through a variety of mechanisms (Wang M. et al., 2023). TMAO can increase the serum levels of the inflammatory cytokine C-C motif chemokine ligand 2 (CCL2) and pro-inflammatory factors (TNF-α, IL-6) in hepatocytes (Rohrmann et al., 2016; Hosseinkhani et al., 2021). Secondly, TMAO can cause intestinal barrier damage and drive macrophage M1 polarization, which in turn aggravates liver inflammation (Nian et al., 2024).

TMAO inhibits the conversion of TC to BAs by activating the FXR-SHP signaling pathway to downregulate CYP7A1 and CYP27A1 expression (Aron-Wisnewsky et al., 2020), which ultimately aggravates lipid accumulation in the liver. The concentration of TMAO has been shown to be correlated with the incidence and severity of NAFLD, as well as with total NAFLD mortality. A cohort study encompassing 5292 subjects (Flores-Guerrero et al., 2021). The study found that serum TMAO levels were positively associated with all-cause mortality in patients with NAFLD, and that TMAO worsened the health status of these patients. However, TMAO did not affect all-cause mortality in non-NAFLD patients. Furthermore, TMAO has been demonstrated to inhibit pancreatic β-cell function, promote β-cell differentiation and apoptosis (Kong et al., 2024), and increase the risk of NAFLD (Rohm et al., 2022). A positive correlation between TMAO content in feces and the degree of hepatic steatosis in mice, which was closely related to the process of pro-inflammatory polarization of macrophages driven by TMAO (Nian et al., 2024). TMAO is a risk factor driving the progression of NAFLD, and inhibiting the synthesis of TMAO can effectively alleviate the development of NAFLD (Corbin and Zeisel, 2012). Consequently, a scientific strategy has been proposed to intervene in NAFLD by inhibiting TMAO synthesis through GM structural remodeling (Arias et al., 2020).

#### 3.2.2 Short-chain fatty acids

Short-chain fatty acids (SCFAs) are metabolites released during the conversion of carbohydrates to monosaccharides by the human GM, mainly including acetate, propionate, and butyrate (Zhang et al., 2019). SCFAs inhibit the progression of NAFLD through the gut-hepatic axis (Chen X.-F. et al., 2020). SCFAs regulate the transcription of key enzymes of hepatic lipid metabolism (FASN, SREBP-1) to influence affect lipid synthesis (Fushimi et al., 2006; Hong et al., 2021). SCFAs have been demonstrated to promote the expression of the rate-limiting enzyme CYP7A1, thereby facilitating the conversion of TC to BA (Guan et al., 2022). In addition, SCFAs have been shown to upregulate the expression of ATP-binding cassette transporter protein A1 (ABCA1). Furthermore, it has been demonstrated that SCFAs enhance the output of TC by up-regulating the expression of ATP-binding cassette transporter proteins G5 and G8 (ABCG5/8), whilst concomitantly inhibiting the expression of ileal Niemann-Pick C1-like 1 (NPC1L1) to reduce TC uptake (He and You, 2020). This ultimately results in a reduction in body lipid accumulation. Butyrate ameliorated hepatic steatosis in HFD-fed mice, this amelioration was closely related to butyrate’s promotion of hepatic ABCA1-mediated cholesterol efflux (Du et al., 2020). In addition, butyrate was found to be associated with the inhibition of intestinal NPC1L1 expression and the upregulation of ABCG5/8 expression in mice (Chen Y. et al., 2018). SCFAs have been shown to improve insulin resistance (IR) by activating G-coupled protein receptors 41/43 (GPR41/43) in the intestine to promote the release of the gastrointestinal peptide hormones tyrosine peptide (PYY) and glucagon-like peptide 1 (GLP-1) from L-cells and reduce lipid intake by suppressing appetite (Psichas et al., 2015; Christiansen et al., 2018).

Furthermore, SCFAs bind to GPR41/43 in the liver, which has been shown to inhibit the expression of lipid-producing genes in the liver by upregulating PPARα expression and activating the AMPK pathway. In addition, this binding has been demonstrated to enhance mitochondrial function to induce the fatty acid oxidation (FAO) of liver fat, thereby increasing lipid consumption, inhibiting liver steatosis, and preventing the development of NAFLD (Hong et al., 2021). SCFAs have also been shown to inhibit cholesterol synthesis in the liver and reduce plasma cholesterol concentrations (Haghikia et al., 2022). Conversely, SCFAs have been observed to downregulate ACC and FASN expression through the AMPK-SREBP-1c pathway, thereby impeding lipid synthesis in hepatocytes (Sun C. et al., 2023). Furthermore, SCFAs have been shown to promote lipid metabolism by increasing CPT1 expression through the PPARα pathway (Kondo et al., 2009). SCFAs have been shown to promote the “browning” of white adipose tissue (WAT) (Sahuri-Arisoylu et al., 2016) and to enhance brown adipose tissue (BAT) thermogenesis and fat oxidation by increasing the expression of peroxisome activator-activated receptor gamma coactivator 1α (pgc-1α) and uncoupling protein 1 (UCP-1) (den Besten et al., 2013). Furthermore, study (Du et al., 2021) has demonstrated that SCFAs can also mediate microRNAs (miRNAs) to regulate gene expression and thereby intervene in the progression of NAFLD, such as via the action of microRNA-378a.

#### 3.2.3 Bile acids

Primary BAs (PBA) are synthesised by the liver from TC and are stored in the gallbladder. Following the ingestion of food, PBA enters the intestine where it is converted into secondary BAs (SBA) by the action of intestinal flora. Of the SBA, 5% is excreted with faeces, while the remaining 95% is reintroduced to the liver via the ileocecal bile acid transporter protein (IBAT), thus forming the enterohepatic cycle of BAs. BAs metabolism has been identified as the predominant pathway of TC consumption in the liver, accounting for 90% of total daily TC consumption (de Aguiar Vallim et al., 2013). BAs biosynthesis has been identified as the major pathway of TC metabolism. An imbalance in cholesterol homeostasis results in intrahepatic TC accumulation, which in turn induces NAFLD, and an imbalance in cholesterol homeostasis is characterised by activation of cholesterol biosynthesis, increase in cholesterol de-esterification, and attenuation of cholesterol export and bile acid synthesis pathways (Henkel et al., 2018). Consequently, the activation of BAs biosynthesis has emerged as a promising therapeutic approach to mitigate NAFLD (Perino et al., 2021). Secondly, accumulated TCs activate Kupffer cells (KCs) and stellate cells (HSCs), triggering mitochondrial dysfunction and endoplasmic reticulum stress, which ultimately drives NAFLD development (Ioannou, 2016). Secondly, BAs have been demonstrated to affect NAFLD by modulating lipid metabolism. In the enterohepatic circulation, BAs primarily influence energy metabolism via the Farnesoid X Receptor (FXR) and G-protein coupled receptor 5 (TGR5) (Fiorucci and Distrutti, 2022). Specifically, FXR has been shown to inhibit hepatic lipid synthesis. Firstly, FXR activation by BAs induces Small Heterodimer Partner (SHP) expression and thus inhibits the activation of SREBP-1c, a key regulator of lipid synthesis genes, to suppress DNL (Adorini and Trauner, 2023). Conversely, FXR has been shown to promote hepatic lipid metabolism. Activation of FXR by BAs induces FGF15/19, which then binds to hepatic FGFR4/β-Klotho, thereby facilitating increased FAO and glucose metabolism (Adorini and Trauner, 2023). In addition, BAs activation of FXR has been demonstrated to promote FAO by inducing PPARα expression (Pineda Torra et al., 2003). Furthermore, FXR has been shown to accelerate cholesterol and triglyceride clearance via Scavenger Receptor Class B Type I (SR-BI), Syndecan-1 (SDC1), and Very Low-Density Lipoprotein Receptor (VLDLR) (Fiorucci et al., 2020). A recent study (Clifford et al., 2021) also found that activation of hepatic FXR by BAs specifically inhibited the expression of lipid synthesis genes Scd1, Dgat2 and Lpin1 in the liver. Notably, this effect was observed to be independent of the FXR-SHP-SREBP-1c pathway. In addition, BAs have been shown to inhibit intestinal absorption of lipids and thereby reduce intrahepatic lipid levels, an effect that is greatly dependent on the intestinal FXR. FXR agonists have been widely used in the treatment of NAFLD, such as obeticholic acid (OCA), and the efficacy of OCA in the treatment of NAFLD has also been demonstrated (Younossi et al., 2019).

Activation of TGR5 by BAs stimulates the secretion of PYY and GLP-1 from intestinal L cells via the cAMP signalling pathway, thereby improving insulin resistance (IR) and suppressing appetite, and consequently reducing lipid intake (Wahlström et al., 2016). Concurrently, activated TGR5 stimulates the process of brown adipose tissue (BAT) thermogenesis and thyroid hormone (T3) production in skeletal muscle, thereby increasing energy expenditure. Furthermore, researchers have demonstrated that activation of TGR5 by BAs acts on the signal-regulated kinase (ERK)/mitochondrial dynamin-related protein 1 (Drp1) pathway, which in turn drives WAT browning as well as increasing FAO (Velazquez-Villegas et al., 2018). A significant increase in conjugated 12α-hydroxylated (12α-OH) BAs, including taurodeoxycholic acid (TDCA) and glycodeoxycholic acid (GDCA), was observed in the livers of patients with hepatic fibrosis and mice (Xie et al., 2021). The combination of 12α-OH BA and TGR5 increased the expression of hepatic fibrosis-related proteins (α-SMA, TGF-β, COL I and PDGF) expression. It is noteworthy that serum BAs was more sensitive to alterations in liver disease than fecal BAs (Chen W. et al., 2020), which also predicts that serum BA may be a better reflection of disease changes than fecal BA.

## 4 Active metabolites in natural products modulate gut microbiota-lipid metabolism communication in non-alcoholic fatty liver disease

The use of natural products as a complementary therapy has garnered increased attention. It is well established that natural products comprise intricate chemical metabolites, with the capacity to act on numerous targets to elicit a therapeutic response. In recent years, there has been a discernible rise in research endeavors exploring the therapeutic potential of natural products in the context of GM-related diseases. This prompts the question of whether natural products can improve NAFLD by targeting and intervening in GM-lipid metabolism. A substantial body of research has already yielded results. As shown in Table 1.

**TABLE 1:** Mechanism of natural products targeting the GM-lipid metabolism in the treatment of NAFLD.

Active metabolites	Natural sources	Experimental model	Dose; Duration; grouping	Regulation of GM and its metabolism	Targeting lipid metabolism	Refs
Resveratrol (RSV)	*Polygonum cuspidatum Sieb. Et Zucc*	6-week-old male C57BL/6J mice	RSV 0.5% in diet; 8 weeks; grouping: (1) LFD group, (2) HFD group, (3) HFR group (HFD + RSV 0.5% in diet)	*Lactobacillus↓, Bifidobacterium↓, Enterococcus↓*	Reduces intestinal SR-B1 protein expression and increases fatty acid β-oxidation	Pang et al. (2023)
		6-week-old male C57BL/6J mice	RSV 300 mg/kg/d; 16 weeks; grouping: (1) NCD group, (2) HFD group, (3) RSV group (HFD +300 mg/kg/d RSV)	*Firmicutes* ↓, *Actinobacteria*↓, *Verrucomicrobia* ↑; *Blautia* ↓, *Lactobacillus* ↓; *Akkermansia Muciniphila* (*A. muciniphila*) ↑; 4-HPA ↑	Activation of the SIRT 1 pathway regulates brown fat and thermogenesis	Wang et al. (2025)
		Male SD rats	RSV 50 mg/kg/d, 100 mg/kg; 6 weeks; grouping: (1) NCD group, (2) HFD group, (3) L-Rsv group (HFD +50 mg/kg/d RSV), (4) H-RSV group (HFD +100 mg/kg/d RSV)	gut microbiota diversity ↑, SCFAs ↑, LPS ↓	No specific mechanism	Chen et al. (2020b)
		5-week-old male C57BL/6J mice	RSV 300 mg/kg; 16 weeks; grouping: (1) SD group, (2) HFD group, (3) HFDR group (HFD +300 mg/kg/d RSV)	SCFA-producing bacteria ↑	No specific mechanism	Wang et al. (2020b)
Curcumin (Cur)	*Curcuma longa* L	6-week-old SD male rat	Cur 100 mg/kg/d, Antibiotic (Abx) comprises vancomycin (0.25 g/L), eomycin sulfate (0.5 g/L), metronidazole (0.5 g/L), ampicillin (0.5 g/L); for 12 weeks; grouping: (1) NASH group, (2) Cur group, (3) NASH + Abx group, (4) Cur + Abx group	gut microbiota diversity ↑, tetrahydrocurcumin (THC) ↑	THC improves the function of LSECs through the NF-κB and PI3K/AKT/HIF-1α signaling pathways, indirectly reducing the fat degeneration and damage of hepatocytes	Wu et al. (2023)
		Human	Cur 500 mg/day; 24 days; grouping: (1) control group (placebo), (2) Cur group (500 mg/kg/d Cur)	*Firmicutes*/*Bacteroidetes* ↓, *Bacteroidetes* ↑	Activated by TGR5 to promote lipid metabolism	He et al. (2024)
		4-week-old SD male rat	Cur 200 mg/kg/d; 4 weeks; grouping: (1) NCD group, (2) HFD group, (3) Cur group (HFD +200 mg/kg/d Cur)	*Blautia* ↑*, Allobaculum* ↑; *Ruminococcus*↓, *Coprococcus*↓, *Mucispirillum* ↓; LPS ↓	Reduces lipid deposition and inhibits liver inflammation	Feng et al. (2017)
Chlorogenic acid (CGA)	*Lonicera japonica* Thunb	C57BL/6 mice; *Fxr−/−* mice	1.34 mg/kg/day CGA, 19 weeks, Grouping: (1) ND, (2) HFD, (3) HFD + CGA, (4) HFD + ADW, (5) HFD + ADW + CGA 1.34 mg/kg/day CGA; 4 weeks; grouping: (1) ND group, (2) HFD group, (3) CGA group (HFD +1.34 mg/kg/d CGA), (4) CGA group (HFD +10 mg/kg/d OCA)	*Bacteroidetes* ↑, *Verrucomicrobia* ↑, *Tenericutes* ↑; *Lachnospiraceae* ↓; *Roseburia* ↓, *Desulfovibrio* ↓	Regulates gut bile acid metabolism, promotes cholesterol metabolism and bile acid excretion regulated by FXR to improve lipid accumulation in the liver	Li et al. (2023)
		6-week-old male C57BL/6J mice	CGA 60 mg/kg/d; 12 weeks; grouping: (1) control group, (2) CGA group, (3) HFD group, (4) CGA + HFD group	*Bifidobacterium* ↑, *Escherichia* ↓	Increases Occulin and ZO-1 tight junction proteins on the intestinal mucosa, reduces the level of inflammatory factors in the serum, and inhibits the activation of the TLR4 signaling pathway	Shi et al. (2021)
		4-week-old male Kunming mice	CGA 200 mg/kg/d, 400 mg/kg/d; 12 weeks; grouping: (1) normal control group (saline 0.4 mL/d), (2) high L-carnitine control group (3% L-carnitine), (3) L-CGA (3% L-carnitine and 200 mg/kg/d CGA), (4) H-CGA (3% L-carnitine and 4200 mg/kg/d CGA)	*Akkermansia* ↑, *Bacteroides* ↑ *Erysipelatoclostridium* ↓, *Faecalibaculum* ↓; TMAO ↓, SCFAs ↑	CGA reverses elevated blood lipids and liver inflammatory factors in mice	Zhang et al. (2021)
		8-week-old male C57BL/6J mice	1.34 mg/kg/d CGA; 4 weeks; grouping: (1) control control group, (2) HFD group, (3) HFD + CGA (geniposide 90 mg/kg/d and CGA 1.34 mg/kg/d), (4) HFD + QHD (10 mL/kg/d), (5) HFD + NaB (200 mg/kg/d)	gut microbiota diversity ↑, tight junction proteins ↑	Reduces signaling of endotoxin and infiltration of Kupffer cells	Peng et al. (2018)
Berberine (BBR)	*Coptis chinensis* Franch	8-week-old male C57BL/6J mice	BBR 200 mg/kg/d; 8 weeks; grouping: (1) NCD group, (2) HFD group, (3) HFD + BBR (200 mg/kg/d)	*Blautia producta* ↑, *Clostridiales bacterium_VE202_06* ↑, *Akkermansia muciniphila* ↑	Upregulates LDLR expression in the liver, promoting the uptake of LDL by the liver	Yang et al. (2022)
		Male beagle dog	BBR 50 mg/kg, 7 days, grouping: (1) BBR by single (50 mg/kg), (2) multiple doses (50 mg/kg/d); 7 days	butyrate-producing bacteria↑	Butyrate enters the bloodstream and exerts a lipid-lowering effect	Feng et al. (2018)
		HepG2 cell	15 μM BBR; 24 h; grouping: (1) control, (2) 15 μm M3 + 2.5 μM PD98059, (4) 15 μm A8 + 2.5 μM PD98059, (4) BBR	No specific mechanism	Inhibit PCSK9 expression	Cao et al. (2019)
Betaine	*Beta vulgaris *L	9-week-old male C57BL/6J mice	LFD for 18 weeks; 18 weeks; grouping: (1) HFD for 18 weeks, (2) HFD 9 weeks + LFD 9 weeks; (3) HFD 9 weeks + (HFD + unprocessed rye bran) 9weeks, (4) HFD 9 weeks + (HFD + bioprocessed rye bran) 9 weeks, (5) HFD 9 weeks + (HFD + unprocessed wheat aleurone) 9 weeks, (6) HFD 9 weeks + (HFD + bioprocessed wheat aleurone), (7) LFD for 18 weeks	*Coriobacteriaceae* ↑; *Akkermansia* ↑, *Bifidobacterium* ↑, *Lactobacillus* ↑, *Ruminococcu* ↑	No specific mechanism	Koistinen et al. (2019)
		9-week-old C57BL/6J mice	1% wt/vol betaine; From the start of pregnancy until the offspring mice reach 3 weeks of age; grouping: (1) standard diet, (2) HFD, (3) HFD +1% wt/vol betaine	*Desulfovibrio* ↓*, Ruminococcus* ↓, *Bacteroides* ↑, *Parabacteroides* ↑, SCFAs ↑	Increases the mRNA expression of Pparα, Cpt1α, and Fatp2 to promote lipid metabolism	Sun et al. (2023b)
Quercetin (Que)	Scutellaria baicalensis Georgi	7-week-old male C57BL/6J mice	Que 100 mg/kg/d; 10 weeks; grouping: (1) normal chow diet, (2) normal chow diet + Que, (3) HFD, (4) HFD + Que	*A.muciniphila* ↑, indole-3-lactic acid (ILA) ↑	FTO/m6A/YTHDF2/CYP8B1 pathway promotes the conversion of TC to BA, which in turn activates FXR to inhibit lipid synthesis	Liu et al. (2025)
		C57BL/6J mice	Que 50 mg/kg/day +100 μL/10 g body weight of 0.15% carboxymethylcellulose sodium; 7 weeks; control group: (1) Con group (carboxymethylcellulose sodium), (2) MetS group (carboxymethylcellulose sodium), (3) MetSQ group (Que)	*Lactobacillus* ↑	Promotes the synthesis of non-12α-hydroxylated BA in serum and stimulates thermogenesis in adipose tissue	Zhu et al. (2024)
		7-week-old male C57BL/6J mice	0.05% quercetin; 16 weeks; grouping: (1) Control, (2) Control + quercetin, (3) HFD, (4) HFD + quercetin	*Clostridia* ↑, *Bacilli* ↑, *Deltaproteobacteria* ↑, *Akkermansia* ↑, *Erysipelotrichi* ↓, *Betaproteobacteria* ↓	Suppresses the expression of genesinvolved in *de novo* lipogenesis	Porras et al. (2017)
Silymarin	Silybum marianum (L.) Gaertn	5-week-old male C57BL/6J	1% Silymarin; 12 weeks; grouping: (1) HFD, (2) HFD + Silymarin, (3) HFD +30–40 μM/kg/day B12	*Akkermansia* ↑, *Blautia* ↑	Activates the liver’s fatty acid degradation pathway, thereby reducing fat production and enhancing fatty acid oxidation	Sun et al. (2023c)
		Huamn	silymarin 103.2 mg/d; 24 weeks; grouping: (1) silymarin group, (2) placebo group	gut microbiota diversity ↑, *Oscillospiraceae* ↑, SCFA ↑	Regulates bile acid metabolism and promotes lipid metabolism	Jin et al. (2024)
Astragalus polysaccharides (APS)	Astragalus membranaceus Bunge	4-week-old male C57BL/6J	4% APS; 12 weeks; grouping: (1) NCD, (2) HFD, (3) HFD +4% APS	*Firmicutes* ↓, *Bacteroidetes* ↑, SCFAs ↑	Inhibits the expression of glucokinase, CD36 and FASN in liver tissue, promotes the expression of CPT-1α and PPAR-α in the liver, and ultimately inhibits FA synthesis and promotes FAO	Hong et al. (2021)
		SPF male SD rat	200 mg/kg/d mAPS extracts; 4 weeks; grouping: (1) control group, (2) HFD group, (3) mAPS gruop, (4) HFD + mAPS group, (5) HFD + BER (300 mg/kg/)	*Firmicutes*/*Bacteroidetes (F/B) ↓, Proteobacteria ↑;* *Epsilonbacteria ↑*	Enhances AMPK and PPAR-α expression and reduces SREBP-1 expression, the SCFA-GPR41/43 signaling pathway	Zhong et al. (2022)
Ginkgo bilobaSeed Polysaccharide (GBSP)	Ginkgo biloba L	8-week-old male C57BL/6J	GBSP 100 mg/kg/d, 200 mg/kg/d; 12 weeks; grouping: (1) Control diet + isotonic saline, (2) HFD + isotonic saline, (3) HFD +100 mg/kg/d GBSP, (4) HFD +200 mg/kg/d GBSP	*Akkermansia* ↑, *Romboutsia* ↑, *Bacteroides* ↑,*Lactobacillus* ↑; SCFAs↑	Activates the AMPK/ACC signaling pathway to produce 3,4-dihydroxyphenylpropionic acid (DHPPA) to inhibit lipid synthesis	Liang et al. (2025)
Tanshinone (Tan)	Salvia miltiorrhiza bunge	(a)*Caenorhabditis elegans*; (b)6-8-week-old male C57BL/6J	(a) Tan 25, 50, 100 μΜ; 25 days; control group: (1) NCD group, (2) HFD group, (3) HFD +100 μΜ Orlistat, (4) HFD + Tan 25 μΜ, (5) HFD + Tan 50 μΜ, (6) HFD + Tan 100 μΜ (b) Salvia miltiorrhiza ethanol extract 15 g/kg/d; 8 weeks; grouping: (1) NCD, (2) HFD, (3) HFD +0.2 g/kg Metformin, (4) HFD + Salvia miltiorrhiza ethanol extract	*Firmicutes* ↓, *Actinobacteria* ↓, *Bacteroidota* ↑, *Verrucomicrobiota* ↑	Upregulates *TFEB* expression and promotes lipid metabolism in the liver	Zheng et al. (2024)
Diammonium glycyrrhizinate (DG)	Glycyrrhiza uralensis Fisch ex DC	4-week-old male C57BL/6J	DG 150 mg/kg on alternate days; 14 weeks; control group: (1) blank control (NCD group), (2) negative control group (HFD + placebo on alternate days), (3) DG group (HFD + DG 150 mg/kg on alternate days)	*Firmicutes*/*Bacteroidetes* ↓; *Lactobacillus* ↑, *Desulfovibrio* ↓; *Ruminococcaceae* ↑, *Lachnospiraceae* ↑; gut microbiota diversity ↑, SCFAs ↑, tight junction proteins↑, goblet cells ↑	No specific mechanism	Li et al. (2018)
Ginsenosides (GS)	Panax ginseng C. A. Mey	6-week-old male C57BL/6J	GS 100 mg/kg, 200 mg/kg; 12 weeks; grouping: (1) ND group (normal chow diet), (2) HFD group, (3) GS-L group (100 mg/kg), (4) GS-H group (200 mg/kg)	*Bacteroidetes* ↑, *Firmicutes* to *Bacteroidetes* ratio (F/B) ↓; *Parabacteroides* ↑, *Akkermansia* ↑, *Helicobacter* ↓; *Muribaculaceae* ↑, *Lachnospiraceae* ↓	Promotes the liver lipolysis gene (Cpt-1a) and inhibits the lipogenesis genes (Srebp-1c, Fas, Acc-1) to improve liver lipid accumulation	Liang et al. (2021)
		8-week-old male C57BL/6J	(a) GS 47.5 mg/kg, GP 466 mg/kg; 7 weeks; grouping: (1) ND group, (2) HFD group, (3) GP group, (4) GS group (b) 0.1 mL/10 g/d; 4 weeks; grouping: (1) HFD group (saline), (2) FGP group (HFD + FMT from GP mice,0.1 mL/10 g/d), (3) FGS group (HFD + FMT from GS mice,0.1 mL/10 g/d)	Sulfurospirillum ↑, *Bacteroides* ↑, Bifidobacterium ↑, SCFAs ↑	Activation of the SCFA-GLP-1/PYY signaling pathway and intestinal gluconeogenesis	Luo et al. (2024)
Platycodin (PD)	Platycodon grandiflorus (Jacq.) A. DC.	8-week-old male C57BL/6J	(a) 375 mg/kg/d, 1125 mg/kg/d; 12 weeks; grouping: (1) ND; (2) ND + PRE-H; (3) HFD; (4) HFD + PRE-H; (5) HFD + PRE-L (b) 0.2 mL/d, 11 weeks; grouping: (1) Don-HFD, (2) Don-HFD + PRE; (3)Rec-HFD; (4)Rec-HFD + PRE	A.*muciniphila* ↑, tight junction protein 1↑, occludin protein gene Ocln↑	Reduces JNK/IRS phosphorylation in the liver and activates the PI3K/PIP3/Akt insulin signaling pathway	Luo et al. (2023)

### 4.1 Phenols

#### 4.1.1 Resveratrol

Resveratrol (RSV)(300 mg/kg/day by gavage, for 16 weeks) is a stilbenoid polyphenol that is enriched in red wine, grapes and pineapple nectar. RSV can ameliorate hepatic steatosis by repairing the HFD-injured intestinal mucosal barrier, decreasing the abundance of harmful bacteria in the GM, and increasing the abundance of SCFA-producing bacteria (Wang P. et al., 2020). RSV (300 mg/kg/day, for 16 weeks) significantly enriches the GM-derived metabolite 4-hydroxyphenylacetic acid (4-HPA) by modulating the structure of GM, which in turn activates the SIRT 1 pathway to modulate adipose tissue browning and thermogenesis to attenuate obesity-associated symptoms and inflammation in HFD-fed mice. Changes in GM include, at the phylum level, decreasing the abundance of *Firmicutes* and *Actinobacteria*, increasing the abundance of *Verrucomicrobia*; at the genus level, inhibiting the HFD-induced reduction of *Blautia* and decreased the relative abundance of *Lactobacillus*; and at the species level, increased abundance of *Akkermansia Muciniphila* (*A. muciniphila*) (Wang et al., 2025). RSV (0.5% in diet, for 8 weeks) has been shown to inhibit FXR-induced SR-B1 protein expression in the mouse intestine by modulating the composition of GM and its bile acid metabolites (Pang et al., 2023). This modulation not only reduces intestinal coeliac secretion but also upregulates the expression of fatty acid FAO-related genes including *Acadm*, *Ehhadh* and *Cpt1a*. Furthermore, RSV (50 mg/kg/day, 100 mg/kg/day,for 6 weeks) has been shown to enhance the synthesis of SCFAs, reduce LPS production, strengthen intestinal barrier integrity, and inhibit intestinal inflammation, thereby ameliorating the progression of NASH by remodeling the GM structure in a study of SD rats induced with HFD (Chen M. et al., 2020). These results suggest that RSV can intervene in the progression of NAFLD through GM and GM metabolites.

#### 4.1.2 Curcumin

Curcumin (Cur), a polyphenolic phytochemical derived from *Curcuma longa* L, has been shown to improve insulin sensitivity, lower blood lipids, and act as an antioxidant (Slika and Patra, 2020). It has been hypothesised that Cur can intervene in the progression of a variety of diseases by modulating the structure of the GM, which in turn intervenes in the progression of several diseases, including NAFLD (Scazzocchio et al., 2020). Supplementation of rats with Cur (100 mg/kg/day for 12 weeks) has been shown to enhance liver sinusoidal endothelial cells (LSECs) function via the NF-κB and PI3K/AKT/HIF-1α signaling pathways, thereby indirectly mitigating hepatic cell steatosis and damage (Wu et al., 2023). An RCT study (He et al., 2024) that included 80 patients with NAFLD. The subjects were randomly divided into two groups and administered Cur (500 mg/kg/d) and placebo, respectively. The duration of the trial was 24 days. In comparison with the placebo, Cur supplementation led to a substantial reduction in liver fat content, BMI, blood lipid levels, and blood glucose levels in patients with NAFLD. The therapeutic effect was associated with the modulation of GM-mediated BAs metabolism and the promotion of BAs receptor TGR5 activation to increase GLP-1 secretion. Furthermore, a separate study (Feng et al., 2017) demonstrated that Cur (200 mg/kg/d, for 4 weeks) reversed the effects of HFD on GM in rats and improved the degree of hepatic steatosis. Cur supplementation has been shown to increase the abundance of SCFA-secreting bacteria (at the genus level), including *Blautia* and *Allobaculum*, while concomitantly inhibiting the growth of bacteria (at the phylum level) associated with the progression of obesity and diabetes, such as *Ruminococcus*, *Coprococcus*, and *Mucispirillum*. Furthermore, Cur has been demonstrated to reduce GM-derived LPS production, thereby promoting the expression of tight junction proteins, occludin and ZO-1, to enhance the intestinal mucosal barrier.

#### 4.1.3 Chlorogenic acid

Chlorogenic acid (CGA) is one of the important active metabolites in *Lonicera japonica* Thunb (Mahboob et al., 2016; Tajik et al., 2017). The present study (Li et al., 2023) evaluated the effects of supplemental CGA (1.34 mg/kg/day for 4 weeks) on NASH mice under various conditions, including NASH mice, antibiotic-treated NASH mice, and *Fxr−/−* NASH mice. The results demonstrated that liver function and lipid levels decreased in NASH mice, while liver function and lipid levels in antibiotic-treated NASH mice and *Fxr−/−* NASH mice remained unchanged before and after CGA intervention. This finding indicates that the depletion of gut bacteria induced by antibiotics can counteract the therapeutic effect of CGA on NASH, thereby suggesting that the efficacy of CGA in treating NASH is contingent upon FXR functionality. The mechanism of action of CGA involves the modulation of intestinal bacterial metabolism, which is associated with alterations in the composition of GM. These alterations include an increase in the abundance of *Bacteroidetes*, *Verrucomicrobia*, and *Tenericutes* at the phylum level, and a decrease in the abundance of *Lachnospiraceae* at the family level. At the genus level, there was a decrease in the abundance of *Roseburia*, and *Desulfovibrio*. Furthermore, CGA increased the expression of FXR, SHP, and BSEP in hepatocytes, thereby promoting FXR-regulated cholesterol metabolism and bile acid excretion, thus enhancing liver function and reducing lipid levels in MASH mice. Additionally, CGA increased BAs excretion, leading to improved hepatic lipid accumulation. In addition, CGA supplementation (60 mg/kg/d for 12 weeks) elevated insulin sensitivity in mice with HFD-induced NAFLD, increased the abundance of *Bifidobacterium* and decreased the abundance of *Escherichia* in GM, and inhibited activation of the TLR4 signalling pathway. This was achieved by increasing the levels of the tight junction proteins Occludin and ZO-1 in the intestinal mucosa and by decreasing the levels of inflammatory factors in the serum (Shi et al., 2021). Other study (Zhang et al., 2021) supplemented mice fed L-carnitine with different doses of CGA (200 mg/kg/d and 400 mg/kg/d). The results demonstrated that, in comparison with the negative control group (supplemented with saline), CGA significantly ameliorated L-carnitine-induced liver damage, including a reduction in hepatitis, steatosis, and oxidative stress. The therapeutic effect of CGA manifested in a dose-dependent manner. CGA exerts its therapeutic effects by inhibiting intestinal TMAO synthesis and reshaping the intestinal microbiota. Their findings revealed that, at the genus level, the abundance of *Akkermansia* and *Bacteroides* significantly increased, while the abundance of *Erysipelatoclostridium* and *Faecalibaculum* decreased in the intestinal microbiota. Intestinal-derived TMAO was reduced and SCFA levels were elevated in the colon. CGA reversed elevated lipids and hepatic inflammatory factors in mice. Furthermore, the administration of CGA (1.34 mg/kg/d) to mice with NAFLD led to an augmentation in the expression of tight junction proteins within the intestinal mucosa. Concurrently, this intervention resulted in the inhibition of tight junction structure degradation and a reduction in the levels of LPS derived from the intestine in NAFLD mice. This effect was achieved via the RhoA/ROCK signaling pathway, thereby intervening in the progression of NAFLD (Peng et al., 2018).

### 4.2 Alkaloids

#### 4.2.1 Berberine

Berberine (BBR) is an isoquinoline alkaloid isolated from Rhizoma Coptidis (*Coptis chinensis* Franch.), which has been shown to have beneficial lipid-lowering properties (Feng et al., 2015). Supplementing HFD-induced mice with BBR (200 mg/kg/d) can selectively act on the beneficial intestinal bacterium *Blautia producta*, which in turn upregulates LDLR expression in hepatocytes to increase hepatic uptake of LDL, and increases the abundance of *Lautia spp*. To stimulate the production of SCFAs, thus lowering TC, and effectively ameliorating HFD-induced hyperlipidemia (HLP) (Yang et al., 2022). BBR has been shown to effectively ameliorate the effects of HFD on hyperlipidaemia by promoting the growth of beneficial butyrate-producing bacteria in the intestinal microflora. These bacteria then enter the bloodstream, where they can exert a lipid-lowering effect (Feng et al., 2018). BBR has also been observed to inhibit the PCSKP and the PCSKF via ERK signalling as well as the ubiquitin-proteasome pathway to inhibit the expression of PCSK9 (Dong et al., 2015; Cao et al., 2019), a liver-derived serine protease that binds to LDLR and contributes to the elevation of serum LDL-C levels (Seidah et al., 2014). BBR has been observed to promote the phosphorylation of AMPK in HepG2 cells, which in turn has been shown to reduce the expression of genes related to lipid biosynthesis, such as *FAS*, *GPAT*, and *ACC*, and consequently reduce blood lipid levels (Cao et al., 2013).

#### 4.2.2 Betaine

Betaine, an alkaloid isolated from the molasses of sugar beets (*Beta vulgaris* L) (Du et al., 2018), has been shown to ameliorate hepatic lipid accumulation in both humans and mice induced by HFD (Abdelmalek et al., 2009). Study (Koistinen et al., 2019) has demonstrated that the supplementation of betaine has been found to increase the abundance of *Coriobacteriaceae* at the family level; at the genus level, it has been found to increase the abundance of *Akkermansia*, *Bifidobacterium*, *Lactobacillus*, and *Ruminococcu*, which has been demonstrated to benefit host health. Researchers (Wu et al., 2020) demonstrated that betaine reduces intestinal damage and intestinal permeability, thereby limiting the entry of intestinal-derived LPS into the systemic circulation, and consequently inhibits the LPS/MAPK/NF-κB signaling pathway release of pro-inflammatory cytokines, including TNF-α and IL-1β, and ameliorate the restriction of IRS-1 and PPARα expression by inflammatory factors, while promoting lipid metabolism as well as attenuating hepatic lipid accumulation (Stienstra et al., 2010; Alipourfard et al., 2019). Supplementation of betaine to mothers not only ameliorated the hepatic lipid accumulation in the mother’s own liver but also attenuated the hepatic lipidosis in the offspring caused by the maternal HFD (Sun L. et al., 2023). The study also examined the process of lipid degeneration in the offspring due to the maternal maternal HFD. This outcome was associated with betaine’s capacity to enhance maternal intestinal flora disruption and augment beneficial intestinal metabolites. This included a decrease in the abundance of *Desulfovibrio*, *Ruminococcus*, and an increase in the abundance of *Bacteroides* and *Parabacteroides*, as well as an increase in the concentration of SCFAs in the feces, without significant changes in the levels of BAs and trimethylamine oxide. These changes have been shown to have a significant impact on the expression of lipid metabolism-related genes in the liver, including increased mRNA expression of *Pparα*, *Cpt1α*, and *Fatp2*.

### 4.3 Flavonoids

#### 4.3.1 Quercetin

Quercetin (QUE) is an important plant metabolite of *Scutellaria baicalensis* Georgi. QUE supplementation has been shown to reduce TG and TC levels in mice fed an HDF, with this effect being dose-dependent (Wang T. et al., 2023). QUE has also been demonstrated to decrease the degree of hepatic steatosis in mice. QUE often requires GM to exert its probiotic function, albeit indirectly. According to the findings of recent research (Liu et al., 2025), the administration of QUE (100 mg/kg/d) to mice maintained on HFD has been demonstrated to have a substantial impact on the enrichment of probiotic *A. muciniphila* in GM. The metabolic product indole-3-lactic acid (ILA) produced by *A. muciniphila* activates the FTO/m6A/YTHDF2/CYP8B1 pathway, which facilitates the conversion of TC to BA. This, in turn, activates FXR, thereby inhibiting lipid synthesis. The study (Zhu et al., 2024) established control group (Con) and metabolic syndrome (MetS) model by subcutaneous injection of saline or sodium glutamate (3 mg/g). The MetS mouse were further subdivided into MetS and MetSQ subgroups, which were administered 0.15% sodium carboxymethylcellulose and QUE (50 mg/kg/d), respectively. Compared with Con group and MetS group, the lipid levels and the degree of hepatic steatosis in MetSQ mice were significantly reduced. Que supplementation has been demonstrated to regulate GM structure, thereby enriching the population of *Lactobacillus*. That has been shown to promote the synthesis of non-12α-hydroxylated bile acids, such as ursodeoxycholic acid and lithocholic acid. These bile acids subsequently bind to TGR5 on adipocytes, thereby activating BAT and inducing WAT browning. This, in turn, enhances thermogenesis mediated by mitochondrial uncoupling protein 1 (UCP1), leading to improvements in metabolic dysfunction. Another study (Porras et al., 2017) found that oral QUE (0.05% (wt/wt)) altered GM, which in turn regulated the expression of genes involved in lipid metabolism, including *Lxrα*, *Srebp-1c*, *Cd36*, *Fabp1*, *C/ebpα*, and *Foxa1*. The study also found that QUE reversed impaired intestinal SCFA synthesis and inhibited TLR-4-mediated hepatic inflammation, which ultimately ameliorated NAFLD. In a randomised study of 41 patients with NAFLD, the study was completed. In a randomised, double-blind clinical trial (Li et al., 2024), patients suffering from NAFLD were treated with QUE (500 mg/day) over a period of 12 consecutive weeks. This treatment resulted in a significant reduction in intrahepatic lipid content.

#### 4.3.2 Silymarin

Silymarin, a flavonolignan metabolite extracted from the seeds of *Silybum marianum* (L.) Gaertn, is composed primarily of the isomers silybin, silydianin, and silychristin. Silymarin has been shown to have lipid-lowering and antioxidant effects, with the potential to improve NAFLD (Saller et al., 2001; Abenavoli et al., 2010; Vargas-Mendoza et al., 2014). A study (Sun W.-L. et al., 2023) revealed that silymarin supplementation for a period of 12 weeks led to enhancements in hepatic lipid metabolism in obese rats. Whole-genome shotgun (WGS) and targeted metabolomics studies on a subset of rat fecal DNA samples demonstrated that silymarin supplementation effectively increased the abundance of *Akkermansia* and *Blautia* in the rat intestinal. Furthermore, silymarin’s lipid-lowering effects were found to be associated with an increase in B12-synthesizing bacteria within the GM. In an RCT (Jin et al., 2024), 83 patients with NAFLD were randomly assigned to two groups, receiving either a placebo or silymarin (103.2 mg/day). Following a 24-week period of observation, the results indicated that silymarin administration led to a reduction in liver stiffness and an enhancement in liver function. Additionally, there was an observed increase in GM’s diversity. Specifically, the abundance of *Oscillospiraceae* in the intestine exhibited a marked increase. This bacterium has been linked to a reduced risk of NAFLD and increased SCFAs production (Zhao et al., 2022; He et al., 2023), suggesting that silymarin may play a role in the management of NAFLD by influencing the composition of the GM.

### 4.4 Polysaccharides

#### 4.4.1 Astragalus polysaccharides

Astragalus Polysaccharides (APS), an active metabolite of *Astragalus membranaceus* Bunge has been shown to be effective in attenuating metabolic disorders induced by HFD, including decreasing the extent of hepatic steatosis, inhibiting body mass gain, and improving insulin resistance (Liu et al., 2020). In order to investigate the mechanism of APS treatment for NAFLD, researchers (Hong et al., 2021) used metagenomic sequencing and non-targeted metabolomics analysis to supplement the effects of APS (4%) on HFD mice. In comparison with the ND group and the HFD group, supplementation with 4% APS significantly altered the GM and metabolites in mice. Including decreasing the abundance of *Firmicutes* and increasing the abundance of *Bacteroidetes* as well as the synthesis of SCFAs. Furthermore, APS has been shown to suppress the expression of glucokinase (GK), CD36, and FASN in hepatic tissues, while promoting the hepatic mRNA expression of CPT-1α and PPAR-α, thereby inhibiting FA synthesis and promoting FAO. In addition, adipogenesis and lipolysis are both reduced by APS, which activates AMPK, upregulates PPAR-α, and downregulates SREBP-1c levels (Sun et al., 2014). Researchers (Song et al., 2024) conducted a study in which they found that APS was degraded to SCFA by GM. This degradation subsequently significantly enhanced intestinal integrity and stimulated GPCR43 expression. The promotion of GPCR43 expression was found to stimulate GLP-1 secretion and inhibit NAFLD progression by controlling blood glucose. Furthermore, APS has been demonstrated to enhance GM diversity, increasing the abundance of beneficial bacteria such as *Dubosiella* and *Monoglobus*, and decreasing the abundance of harmful bacteria such as *Escherichia* and *Acinetobacter*. Furthermore, the study (Zhong et al., 2022) administered 200 mg/kg/day of mAPS extracts to HFD mice. The results demonstrated that, in comparison with mice subjected to model group mice (only fed HFD), the administration of mAPS extracts significantly mitigated hepatic lipid accumulation and inflammation, as well as reduced blood lipid levels, induced by an HFD. The results also indicated that supplementation with mAPS extracts enhanced the expression of AMPK and PPAR-α, and reduced the expression of SREBP-1. Furthermore, the therapeutic effects of mAPS extracts were associated with the SCFA-GPR41/43 signaling pathway. In addition, mAPS extracts were found to remodel GM, including at the phylum level, where the application of mAPS extracts resulted in an increase in the abundance of *Proteobacteria* and a decrease in the ratio of *Firmicutes* to *Bacteroidetes*. At the class level, the abundance of *Epsilonbacteria* also exhibited a significant increase.

#### 4.4.2 Ginkgo biloba seed polysaccharide

Ginkgo seeds have a long history in both medicine and food production. Ginkgo biloba (*Ginkgo biloba* L) Seed Polysaccharide (GBSP) is a polysaccharide that is isolated and purified from ginkgo seeds. In murine models of non-alcoholic fatty liver disease (NAFLD), administered at doses of 100 or 200 mg per kilogram of body weight, GBSP has been observed to attenuate liver steatosis, a condition characterized by an accumulation of fat in the liver, induced by HFD (Liang et al., 2025). This effect appears to be the result of a multifaceted regulatory mechanism involving several pathways. GBSP has been shown to significantly increase the abundance of *Akkermansia*, *Romboutsia*, *Lactobacillus*, and *Bacteroides*, as well as to activate the AMPK/ACC signaling pathway, thereby inhibiting lipid synthesis through the production of 3,4-dihydroxyphenylpropionic acid (DHPPA).

### 4.5 Terpenoids

#### 4.5.1 Tanshinone

In the domain of traditional Chinese medicine, *Salvia miltiorrhiza* bunge (Dan shen) has a long history of utilization as an herbal remedy for the treatment of NAFLD. A pivotal metabolite of this medicinal approach is tanshinone (Tan), a crucial active metabolite in Danshen. The knockout of the *Tfeb* gene has been demonstrated to induce lipid accumulation in adipocytes. The present study (Zheng et al., 2024) investigated the effects of Tan at varying concentrations (25 μM, 50 μM, 100 μM) on *Caenorhabditis elegans* (*C. elegans*) induced by HFD. Model group (fed HFD), blank control (normal diet), and positive control (HFD and 100 μM Orlistat) were established. The results demonstrated that Tan induced nuclear translocation of the TFEB homolog HLH-30 in *C. elegans* and reduced fat accumulation, with the lipid-lowering effect of 100 μM Tan being comparable to that of the positive control. Subsequently, researchers administered an ethanol extract of Salvia miltiorrhiza (primarily Tan) to HFD mice via oral gavage (15 g/kg/day for 2 weeks) and established three groups: model group (HFD), blank control group (normal diet), and positive control group (HFD and 0.2 g/kg Metformin). The extract containing abundant Tan has been shown to reduce the abundance of *Firmicutes* and *Actinobacteria*, and increase the abundance of *Bacteroidota* and *Verrucomicrobiota*, thereby improving lipid accumulation in the liver (Zheng et al., 2024). That suggests that Tan treatment for NALFD may be a viable option.

The study (Shou et al., 2025) found that free cholesterol (FC) has a significant impact on the severity of NAFLD, exacerbating the accumulation of triglycerides in the liver by increasing ROS levels, damaging lysosomes, and inhibiting lipophagy. Dihydrotanshinone I (DhT) is a prominent metabolite of tanshinones. Supplementation with DhT has been shown to reduce liver lipids in mice with NAFLD. However, the knockdown of liver *Pparα* negated this effect, and no significant changes in GM or metabolites were observed before or after the intervention with DhT. This observation indicates that DhT activates PPARα pathway, leading to a reduction in ROS, which in turn promotes lipophagy. Notably, this effect is observed to be independent of GM.

#### 4.5.2 Diammonium glycyrrhizinate

Diammonium glycyrrhizinate (DG) is a triterpene saponin metabolite that is extracted from the root of *Glycyrrhiza uralensis* Fisch ex DC. It is currently a first-line pharmaceutical agent for the treatment of inflammation and protection of the liver (Sun et al., 2019). The study (Li et al., 2018) randomly divided normal mice into three groups: blank control group (fed normal diet), negative control group (fed HFD and placebo), and DG group (fed HFD and 150 mg/kg DG). After 2 weeks, the DG group exhibited a marked decrease in body weight, as well as hepatic steatosis and inflammation, when compared with the placebo group. These enhancements are attributable to DG’s capacity to enhance gut microbiota diversity in GM. The observed alterations encompass a decline in the *Firmicutes*/*Bacteroidetes* ratio at the phylum level. At the genus level, the relative abundance of probiotics such as *Lactobacillus* increased, while the relative abundance of LPS-producing bacteria such as *Desulfovibrio* decreased. At the family level, there was an increase in bacteria producing SCFAs, including *Ruminococcaceae* and *Lachnospiraceae*. Concurrently, DG has been observed to facilitate the restoration of intestinal mucosal barrier function by augmenting the expression of tight junction proteins and goblet cells, while concurrently stimulating mucin secretion.

### 4.6 Saponins

#### 4.6.1 Ginsenosides

In traditional Chinese medicine, *Panax ginseng* C. A. Mey is regarded as the “king of botanical drugs”, and ginsenosides (GS) have been characterized as important metabolites of *Panax ginseng* C. A. Mey (Yin et al., 2021). In the study (Luo et al., 2024), the isolation of ginsenosides (GS) and ginsenoside polysaccharides (GP) from dried ginseng slices was conducted. Two groups of mice were fed HFD and supplemented with GS (47.5 mg/kg/d) or GP (466 mg/kg/d), respectively. In comparison with the mice subjected to HFD, supplementation with GS or GP effectively intervened in the development of obesity induced by an HFD. In order to investigate the hypothesis that GS intervenes in obesity through GM, researchers transplanted GM from GS-supplemented mice into another group of mice subjected to an HFD. A blank control group was set up for comparison. The results (Luo et al., 2024) demonstrate that GS and GP can intervene in obesity induced by HFD, and this effect is mediated by GM. Specially GS has been demonstrated to selectively enrich species such as *Sulfurospirillum*, *Bacteroides*, and *Bifidobacterium* within the intestinal tract. Concurrently, *Bacteroides* and *Bifidobacterium* have been observed to facilitate the synthesis of SCFAs. Furthermore, GS has been shown to enhance obesity by stimulating the SCFA-GLP-1/PYY signaling pathway and intestinal gluconeogenesis. In the present study (Liang et al., 2021), the effects of ginsenoside extract on HFD-induced hepatic steatosis and metabolic endotoxemia in mice were investigated. Mice were divided into three groups: normal diet (ND) group, HFD group, and experimental group that received different doses of ginsenoside extract (100 mg/kg or 200 mg/kg). Compared with ND mice and HFD mice, ginsenoside extract significantly alleviated HFD-induced hepatic steatosis and metabolic endotoxemia, and improved liver function and intestinal barrier function. The therapeutic effect exhibited a dose-dependent relationship. This therapeutic effect is attributable to the influence of ginsenoside extract on GM. At the phylum level, ginsenoside extract led to a significant increase in the abundance of *Bacteroidetes* and a concomitant reduction in the *Firmicutes* to *Bacteroidetes* ratio (F/B). At the genus level, ginsenoside extract has been shown to promote the proliferation of *Parabacteroides* and *Akkermansia*, which synthesize SCFAs and regulate metabolic disorders, while inhibiting the prevalence of harmful bacteria *Helicobacter*. At the family level, ginsenoside extract has been shown to promote the prevalence of beneficial bacteria *Muribaculaceae*, while reversing the increase of harmful bacteria *Lachnospiraceae*. Concurrently, ginsenoside extract mitigates liver inflammation by impeding the activation of the NF-κB/IκB signaling pathway. GS has also been observed to enhance liver lipid accumulation by promoting the expression of genes involved in liver lipolysis (*Cpt-1a*) and inhibiting the expression of genes associated with lipogenesis (*Srebp-1c*, *Fas*, *Acc-1*) (Fang and Judd, 2018).

#### 4.6.2 Platycodin

In East Asia, *Platycodon grandiflorus* (*Platycodon grandiflorus* (Jacq.) A. DC.) applications include use as both food and medicine. Platycodin D (PD) is a triterpene saponin metabolite extracted from the roots of *Platycodon grandiflorus*. For NAFLD, the potential of PD to reduce the risk of disease by promoting bile acid biosynthesis has been demonstrated (Kim et al., 2024). Secondly, PD has been shown to improve liver steatosis by downregulating the expression of intestinal lipid uptake proteins (CD36, NPC1L1, and ApoB) (Tang et al., 2024) and upregulating the expression of hepatic lipolysis proteins (CPT1, HSL, and UCP2) (Hwang et al., 2019).

Conversely, PD has been shown to inhibit excessive gluconeogenesis induced by HFD through the AMPK-PCK1-G6Pase signaling pathway, activate the AMPK-ACC-CPT-1 signaling pathway to reduce fatty acid biosynthesis, and increase fatty acid oxidation to reduce liver lipids (Shen et al., 2023). *In vitro* experiments have also demonstrated the protective effect of PD on NAFLD, which may involve reducing the level of oxidative stress induced by palmitic acid in AML-12 cells and enhancing mitochondrial function (Wen et al., 2022). PRE (*Platycodon grandiflorus* root extract) is an extract derived from the decoction of *Platycodon grandiflorus*, containing multiple Platycodin metabolites (Platycodin C, D2, D3, J). A study (Luo et al., 2023) used different doses of PRE (375 mg/kg or 1125 mg/kg) to intervene in mice fed HFD. The results demonstrated that both low and high doses of PRE effectively alleviated symptoms of MetS induced by HFD, including reducing the severity of hepatic steatosis, lowering lipid levels, and improving insulin sensitivity. In order to validate the therapeutic effect of PRE and its relationship with GM, researchers transplanted intestinal microbiota from PRE-treated mice into pseudo-germ-free mice (previously treated with antibiotics) induced by HFD. The results demonstrated that the therapeutic efficacy of PRE in MetS is closely associated with GM. PRE has been demonstrated to promote the enrichment of *A. muciniphila*, thereby activating the downstream PI3K/PIP3/Akt insulin signaling pathway and improving MetS. MetS is a key risk factor for the progression of NAFLD. It is imperative to acknowledge that the experimental design employed PRE, not PD alone. Although UPLC-LTQ-Orbitrap MS analysis indicated that PD was dominant in PRE, the contribution of PD to the therapeutic effects of PRE requires further investigation.

## 5 Discussion and perspective

Despite the fact that the pathogenesis of NAFLD has not yet been elucidated, some progress has been made in the study of its pathogenesis. There is a broad consensus in the scientific community that lipid metabolism disorders play a pivotal role in the development of NAFLD, as evidenced by the traditional “second-strike” theory and the current mainstream “multiple-strike” theory (Santos-Baez and Ginsberg, 2021). Consequently, addressing lipid metabolism disorders is imperative for the treatment of NAFLD.

GM, a complex microbial ecosystem within the human body, influences the balance between disease and illness. The present study established that GM and its metabolites interact with hepatic lipid metabolism through the “gut-liver axis” (Han et al., 2023). This finding suggests that GM and its metabolites may have a role in the prevention and intervention of NAFLD. It is important to note that GM is highly sensitive to external factors, and a variety of metabolites, including those of natural origin, can trigger dynamic changes in GM structure and function. Consequently, the promotion of beneficial GM remodeling through therapeutic interventions holds considerable promise in the treatment of NAFLD. Probiotics have been shown to possess NAFLD therapeutic capabilities by modulating lipid metabolism, inhibiting inflammatory responses, and maintaining intestinal mucosal barrier integrity (Ji et al., 2019).

**FIGURE 3:**
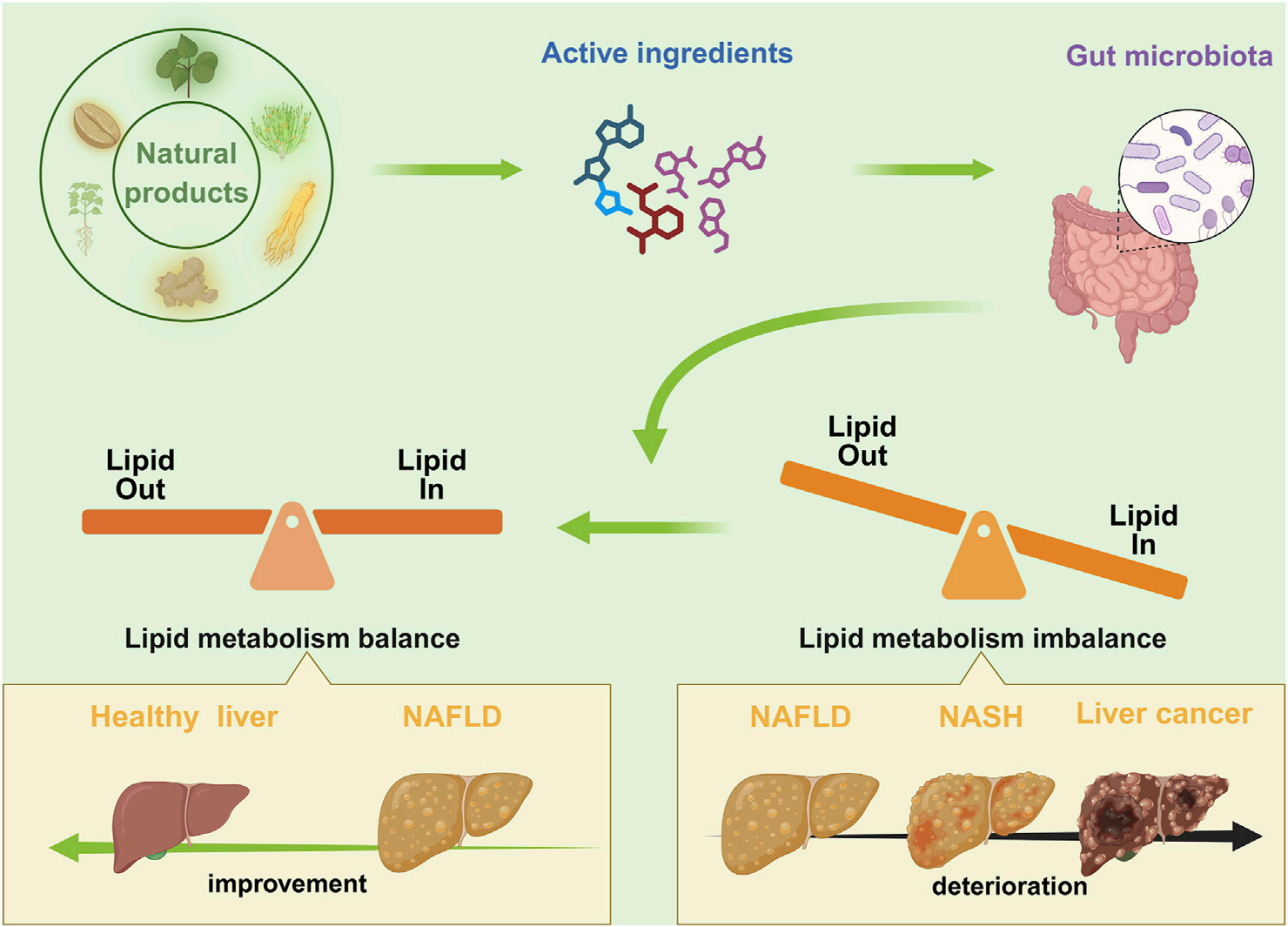
An overview of the therapeutic effects of natural products on NAFLD through regulation of glucose and lipid metabolism. Imbalances in liver lipid metabolism (the accumulation of lipids in the liver exceeds their consumption) can trigger the progression of NAFLD. Natural products regulate liver imbalances through the gut microbiota, promoting lipid metabolism balance in the liver, which helps reverse the progression of NAFLD. NAFLD, non-alcoholic fatty liver disease; NASH, non-alcoholic steatohepatitis.

However, it is important to acknowledge the limitations of studies examining direct probiotic transplantation as a treatment for NAFLD. Firstly, the specific molecular biological mechanisms by which probiotics affect lipid metabolism in the pathogenesis of NAFLD have not been fully elucidated. Secondly, significant inter-individual differences in GM, colonization resistance of host intrinsic flora to foreign probiotics, and survival stability of transplanted probiotics act as obstacles to direct probiotic supplementation. Conversely, natural products have been shown to promote natural growth by supplying the nutrients required for the proliferation of host-intrinsic probiotics, thereby reducing interference with the GM ecosystem (Guo et al., 2022). This approach is hypothesized to be safer and to exhibit reduced colonization resistance. These findings (Koistinen et al., 2019; Yang et al., 2022; Sun W.-L. et al., 2023; Zhu et al., 2024) also confirm the therapeutic efficacy of natural product-derived metabolites in NAFLD. Consequently, the utilisation of natural products to indirectly modulate GM structure and thereby intervene in NAFLD emerges as a potential option. As shown in Figure 3.

Nevertheless, contemporary research endeavors concerning the impact of natural products and their metabolites on hepatic lipid metabolism through GM and its metabolites continue to encounter significant challenges. First, there is a paucity of studies that specifically examine the molecular mechanisms by which GM influences hepatic lipid metabolism. Secondly, the synthesis mechanisms of GM metabolites remain unclear, and the mechanisms by which natural product derivatives influence GM metabolites also require further elucidation. It is imperative to investigate the mechanisms by which GM metabolites regulate the expression of genes associated with lipid metabolism through epigenetic modifications. Furthermore, there is a necessity to explore methodologies that can be employed to overcome the heterogeneity in treatment efficacy that is caused by differences in GM composition among individuals. A significant challenge in the field pertains to the limited water solubility, poor intestinal absorption rates, and substantial first-pass metabolism exhibited by numerous metabolites of botanical drugs, which collectively result in considerably diminished bioavailability. A major challenge in this field is the heterogeneity of the studies, which is characterized by significant variations in dosage, treatment duration, and evaluation criteria. This heterogeneity poses a significant obstacle to the comparison of results across studies. In order to address these challenges, researchers must collect large-scale gut microbiome data and combine it with multi-omics technologies to analyze the potential associations between GM characteristics and lipid metabolism. Furthermore, subsequent studies ought to prioritize the investigation of the particular mechanisms that govern the interactions between GM and lipid metabolism within the body. This will facilitate the development of precise strategies for modulating lipid metabolism through GM reprogramming. Such research not only reveals the impact of individual gut microbiome differences on health status and treatment sensitivity but also assists clinicians in developing personalized treatment plans based on individual GM profiles and lipid metabolism states. Furthermore, advancements in technology have led to innovations that enhance bioavailability. These include the utilization of liposomes, polymer nanoparticles, and other encapsulation technologies to improve solubility and targeting, as well as conducting structural modifications to enhance stability and absorption rates. These technological advancements have proven effective in improving bioavailability.

In conclusion, natural products hold considerable promise in the treatment of diseases and merit further investigation. Natural products have been shown to regulate lipid metabolism in NAFLD by affecting GM and metabolites, thereby intervening in the progression of NAFLD. The potential of natural products in driving NAFLD therapy by targeting GM and lipid metabolism is significant, as it not only enriches the theoretical underpinnings of the gut-hepatic axis, but also deepens our understanding of natural products.
